# Probabilistic grammatical model for helix‐helix contact site classification

**DOI:** 10.1186/1748-7188-8-31

**Published:** 2013-12-18

**Authors:** Witold Dyrka, Jean‐Christophe Nebel, Malgorzata Kotulska

**Affiliations:** 1 Institute of Biomedical Engineering and Instrumentation, Wroclaw University of Technology, Wroclaw, Poland; 2 Inria Centre de Recherche Bordeaux Sud‐Ouest, Talence, France; 3 Faculty of Science, Engineering and Computing, Kingston University, London, UK

**Keywords:** Probabilistic context‐free grammar, Grammar inference, Genetic algorithm, Helix‐helix contact, Protein structure prediction

## Abstract

**Background:**

Hidden Markov Models power many state‐of‐the‐art tools in the field of protein bioinformatics. While excelling in their tasks, these methods of protein analysis do not convey directly information on medium‐ and long‐range residue‐residue interactions. This requires an expressive power of at least context‐free grammars. However, application of more powerful grammar formalisms to protein analysis has been surprisingly limited.

**Results:**

In this work, we present a probabilistic grammatical framework for problem‐specific protein languages and apply it to classification of transmembrane helix‐helix pairs configurations. The core of the model consists of a probabilistic context‐free grammar, automatically inferred by a genetic algorithm from only a generic set of expert‐based rules and positive training samples. The model was applied to produce sequence based descriptors of four classes of transmembrane helix‐helix contact site configurations. The highest performance of the classifiers reached *AUCROC* of 0.70. The analysis of grammar parse trees revealed the ability of representing structural features of helix‐helix contact sites.

**Conclusions:**

We demonstrated that our probabilistic context‐free framework for analysis of protein sequences outperforms the state of the art in the task of helix‐helix contact site classification. However, this is achieved without necessarily requiring modeling long range dependencies between interacting residues. A significant feature of our approach is that grammar rules and parse trees are human‐readable. Thus they could provide biologically meaningful information for molecular biologists.

## Background

Language is a way in which an infinite number of meanings can be expressed by a finite number of symbols using a finite number of rules. Strikingly, this creativity is a feature shared by natural languages and languages of nature [1]. Indeed, numerous sequences and folds of polymeric biomolecules, such as DNA, RNA and proteins, are built from just a few types of nucleotides and amino acids. Moreover, physicochemical laws that drive folding and determine function of biopolymers can be sometimes confined to rules as simple as Watson‐Crick pairing. Since the 1980s methods of computational linguistics have been applied to molecular biology [2]‐ [7], gradualy gaining importance in the field [1,8]‐ [11]. For example, probabilistic context free grammars have become a standard approach to RNA structure prediction [12]‐ [18]. In the realm of proteins, non‐probabilistic regular expressions are used to represent functional patterns in PROSITE [19,20], while Hidden Markov Models power state‐of‐the‐art homology search tools such as HHsearch [21,22], HHblits [23] and HMMER [24]‐ [26], and are used to represent Pfam domains [27,28]. While excelling in their tasks, these methods of protein analysis do not convey directly information on medium‐ and long‐range residue‐residue interactions. As these interactions determine protein structure, they are essential for defining a *protein language*. This requires an expressive power of at least context‐free grammars. However, application of more powerful grammar formalisms to protein analysis has been surprisingly limited. They have mainly been concerned with modeling of *β*‐sheets [29]‐ [34] and *α*‐helical pairs and bundles [35,36] using context‐free and mildly context‐sensitive grammars. Moreover, probabilistic context‐free grammars have been applied to analysis of ligand binding sites [37] and antimicrobial peptides [38] showing that produced parse trees could provide biological insight into the modeled structures. Finally, whatever their complexity level, grammatical models have proved to be useful for protein structure annotation [37,39] and prediction [32,35,40]‐ [42] and to designing synthetic peptides [38,43].

In this work, we present a probabilistic grammatical framework for *problem‐specific* protein languages and apply it to classification of transmembrane helix‐helix pairs configurations. The model covers the lexical (primary structure) and syntactical (secondary and tertiary structure) levels of protein linguistics. Moreover, as protein function cannot be separated from protein structure, our model also reaches the semantic level.

### Protein structure prediction from intra‐protein contacts

Transmembrane (TM) proteins are important targets for computational modeling methods, as they are ‐ despite recent progress ‐ significantly underrepresented in the Protein Data Bank [44]. It has been estimated that around 25‐30% of proteins in human body are TM proteins [45,46]. Unfortunately, since TM proteins are usually very large water insoluble molecules anchored in the lipid bilayer, their extraction, crystalization and analysis are difficult tasks. Currently only 2% of structures stored in PDB belong to transmembrane proteins, according to PDBTM service [47], as of April 2012. The lack of experimental structures cannot be compensated by template‐based modeling (homology and threading), which is estimated to cover no more than 10% of all human TM proteins [46].

Widely‐used de novo approaches to structure prediction usually rely on exploration of protein conformational space by utilizing existing knowledge (such as database of fragments), and evaluation of candidate solutions by minimizing energy functions [45,48]‐ [52]. Successful predictions by these methods are currently limited to proteins up to 200‐300 amino acids long because computational power limits the size of the conformational phase space that can be searched, typically 20,000‐200,000 models per protein [53,54]. It was suggested that prediction of larger protein domains would become possible upon introduction of additional constraints to the conformational space [55], such as accurately predicted residue contacts [56,57]. Since contacts between distant residues tend to determine the overall global protein structure, prediction of these molecular contacts has been recognized early as a promising strategy in predicting the three‐dimensional structures of proteins [48,58]‐ [60]. It was estimated that as few as one contact in every eight residues would be sufficient to find the correct fold of a single domain protein [59,61]. In a recent study, Sathyapriya *et al.*[62] have shown that it is possible to reconstruct a protein model at 4.8 Å resolution based on a partial contact map containing only 8% of all contacts. This research, which selected key contacts using a graph theory algorithm, suggests that some contacts are structurally more significant than others. They called them the structural essence of protein, since they seem crucial for protein core packing. Interestingly, this structural essence contains mostly inter‐secondary structural contacts and contacts from loop regions, while ignoring intra‐secondary structural contacts and contacts on the protein surface. Moreover, even the prediction of a few contacts is useful to constrain conformational searches in ab initio prediction [53,63]. Using inter‐residue contact information in the process of protein modeling can be beneficial at multiple stages, from adding constraints during the folding process, through refinement of produced models, to ranking of final models [54,64]. Consequently, the prediction of intramolecular contacts has become an active field of research. Intramolecular contact prediction methods can be classified into three non‐exclusive categories [65]: homologous template, machine learning [65]‐ [70] and statistical correlated mutations [71]‐ [75] approaches. Recently, Hopf *et al.*[46], and Nugent and Jones [76] have shown that, by applying contact information extracted from evolutionary covariation of amino acids [77,78], the upper limit of the size of predicted TM protein of unknown structure could be lifted up to at least 500 residues. Remarkably, this required only ca. 500 candidate models per protein and no use of database fragments [46,79]. However, predicted residue contacts required validation by predicted TM topology [46]. Moreover, these evolutionary covariation methods [77,78] rely on availability of hundreds of homologous sequences.

### Helix‐helix contacts in transmembrane proteins

Over 80% of known structures of transmembrane (TM) proteins are classified as *α*‐helical [47]. Despite a wide variety of biological functions, they display relatively simple architecture [80]. In TM proteins, molecular contacts between helices are crucial as they provide a structural skeleton. TM helices are typically longer (on average: 26 amino acids) and more tightly packed than helices in soluble proteins [81,82]. A stable interaction between two helices requires that several residues from each helix are involved in the helix‐helix contact [83]. We call this structure a helix‐helix interface in this manuscript.

Despite a relatively small number of experimental structures, substantial research effort during the last decade revealed many distinctive features of TM helix‐helix interactions. Similarily to water soluble proteins, four residues, i.e. leucine, alanine, isoleucine and valine, mediate helix interactions in a coiled‐coil fashion. They form a *knobs‐into‐holes* motif represented by the leucine zipper: a heptad repeat of leucine residues, LxxLxxxLxx [84,85]. In addition, a second motif GxxxG containing tightly packed small residues, alanine, glycine, serine and threonine is characteristic of transmembrane proteins [85,86]. Indeed, the side chains inside helix‐helix interfaces on average are shorter than those in the non‐interface parts of the helices [87]. Interestingly, the glycine and proline residue types, normally associated with helix‐breaking propensity, are relatively common in transmembrane helices [88]. This suggests that glycine residues serve as molecular notches for orienting multiple helices in protein complexes [89]. Recently, Marsico *et al.*[90] developed a database of sequential motifs in transmembrane proteins using a structural fragment clustering method. While they found 213 statistically significant non‐redundant motifs specific to *α*‐helical transmembrane proteins, only 5 of them were assigned a role in helix‐helix packing.

Helices have smaller crossing angles in membrane proteins than in water soluble proteins, which is consistent with the requirements of structural compactness and membrane spanning [88]. The helix‐helix packing mode in transmembrane proteins was quantitatively studied by [83]. They represented the mutual orientations of local axes by a single parameter. It was showed that a specific range of parameter values is preferred for helices of anti‐parallel orientation. However, interactions between helices of parallel orientation appeared to be less constrained. Finally, a study by Walters and DeGrado [91] on helix packing motifs revealed that 90% of known configurations of helix‐helix interactions in transmembrane proteins could be accurately represented using only a set of eight 3D templates [91]. In their research, helix pairs were clustered according to the 3D similarity (RMSD ≤ 1.5 Å) of their fragments involved in the helix‐helix contact. Their study also highlighted position‐specific sequence propensities of amino‐acids and the occurrence of the GxxxG motif.

### Helix‐helix contact prediction in transmembrane proteins

An early approach to helix‐helix interaction prediction was based on a scoring function which rewarded the formation of contacts between small residues and penalized the burial of large amino acids [92]. A method relying on co‐evolving residues was developed by Fuchs *et al.*[73]. Using a consensus approach combining correlations from different algorithms, 53% of predicted contacts were within one helix turn from the observed contacts. Moreover, 72% accuracy was reported for helix‐helix contact prediction based on at least 5 correlated mutations per helix pair. The same group predicted contacts between residues with 26% accuracy using a neural network approach [69]. This resulted in 78% accuracy in helix‐helix contact prediction. Lo *et al.*[93] developed the webserver TMhit, which predicts membrane protein topology using a support vector machine. Later, they designed a framework for prediction of helix‐helix interactions in membrane proteins from residue contacts, which achieved 56% accuracy [68]. Recently, Nugent and Jones [70] trained a support vector machine classifier that predicted residue contacts and helix‐helix interactions in TM proteins. They reported an accuracy of up to 70% per residue‐residue contact and up to 65% per helix‐helix interaction, slightly better than a similar method called SVMcon [67]. In another line of research, Barth *et al.*[53] addressed the problem of helix‐helix interaction prediction by creating sequence profiles from a library of helix pairs whose spatial configurations were known. In their method a helix pair in the query was compared to helix pairs in the library by calculating profile‐profile scores between the pairs. While the overall accuracy of helix packing prediction was rather low (17‐30% of correct backbone orientations), it was sufficient to constrain ab initio prediction of TM protein structures in ROSETTA [45,49,50]. Significantly, this approach does not model interactions between contacting residues from the two helices since this would require a more complex model than sequence profiles.

In general, the accuracy of methods for helical transmembrane proteins topology prediction appears to be higher than most prediction methods applied on globular proteins, however it decreases slightly with the increasing variety of structures [94]. Unfortunately, the most successful machine learning techniques do not reveal which biophysical or biochemical features of interacting helices are relevant to achieve high level of accuracy [94]. Such a method would require the ability to represent long range dependencies between contact residues crucial for helix‐helix interactions. Waldispuehl and Steyaert [35] proposed a multi‐tape S‐attributed grammar to represent helix bundles in TM proteins. In their model, a single pair of helices in an *α*‐helix bundle is described by a set of grammar rules of a non‐probabilistic context‐free language capable for accounting of majority of inter‐helical dependencies. At each stage of the sequence processing, a value (attribute) that reflects folding cost is calculated.

Sequence‐based single helix prediction of transmembrane proteins has already achieved a very satisfactory level, i.e. 97% accuracy per residue [95]. Moreover, since the accuracy of helix‐helix contact site prediction based on intra‐protein contact prediction has recently reached 70‐80% [67,69,70,73], we propose to extend these previous studies. In this work, we introduce a probabilistic context‐free grammar‐based method for sequence‐based prediction of structural classes of transmembrane helix‐helix contact sites. The specific task addressed in this research is to predict the contact site class of a helix‐helix pair assuming that the helix pairing is known (e.g. the i‐th helix is in contact with the j‐th one). The classifier predicts whether the pairing belongs to a particular structural class of helix‐helix contact site. Our clustering of transmembrane helix‐helix contact sites into structural classes is based on the pioneering work of Walters and DeGrado [91]. According to their research, 67% of interacting helix pairs can be grouped into only 4 main classes. While their classification was based purely on a geometrical basis, we worked on the hypothesis that there are sequence level motifs associated to these classes, analogously to patterns described in [85,86,90], hence our method is only sequence‐based. Since a helix pair representative for a structural class can be regarded as a 3D template, our task can be interpreted as assigning the appropriate 3D template to a contact site between a pair of helices of unknown 3D structure. Hence, this is equivalent to prediction of 3D structure of protein fragments. Such a method could be valuable to constrain ab initio protein structure predictions or for threading refinement.

## Methods

### Probabilistic formal languages

A language *L* is usually specified by a grammar *G*, which is denoted as *L*=*L*(*G*). Classical grammar formalism, derived from Noam Chomsky’s early research in computational linguistics [96], defines a grammar *G* as a tuple:

(1) G=<Σ,N,P,S>,

where *Σ* is a finite set of terminal symbols (alphabet), *N* is a finite set of non‐terminal symbols, *P* is a finite set of production rules, and *S*∈*N* is a start symbol. *N* and *Σ* are mutually disjoint. Terminal symbols (or simply terminals) are the only accepted symbols to appear in a final sentence generated by a grammar, whilst non‐terminal symbols (or non‐terminals) are used as temporary symbols by a procedure of sentence derivation. All production rules are in the form:

(2) $${\left(\Sigma \cup N\right)}^{*}N{\left(\Sigma \cup N\right)}^{*}\to {\left(\Sigma \cup N\right)}^{*},$$

where ∪ denotes a set union and (*Σ*∪*N*)^∗^ is the set of all strings over symbols in *Σ*∪*N*, including the empty string.

Grammars can be classified on the basis of restrictions imposed on their production rules, which determine expressive power of the generated language. A context‐free grammar is able to generate sentences with nested dependencies. Context‐free languages are generated using rules with only one Left Hand Site (LHS) non‐terminal and any combination of terminal and non‐terminal symbols on the Right Hand Site (RHS):

(3) $$N\to {\left(\Sigma \cup N\right)}^{*},$$

This formal definition of a non‐probabilistic context‐free grammar *G*=<*Σ*,*N*,*P*,*S*> restricts each rule from the set *P* to the following form:

(4) A→α,

where *A*∈*N* and *α*∈(*Σ*∪*N*)^∗^[97]. The computational complexity of recognition whether a certain sentence belongs to a given context‐free language is polynomial (*O*(*n*^2.81^) [97,98]) in time in respect to the length of the sentence [99]‐ [103].

Probabilistic formal language *L* is a generalisation of the non‐probabilistic formal language concept in the probabilistic domain [104]. A probabilistic language can be viewed as a probability distribution, *P*(*X*|*L*), over a set of sentences *X*. Consequently, *P*(*x*|*L*) is the conditional probability of drawing a sentence *x* given language *L*. Probabilistic formal grammar *G* is a description of *P*(*X*|*L*(*G*)), which is usually abbreviated to *P*(*X*|*G*).

A *probabilistic context‐free grammar* (PCFG) is defined similarly to a non‐probabilistic CFG, where probabilities Pr are attributed to each rule:

(5) A→α,Pr(A→α).

A probabilistic context‐free grammar is *proper* if

(6) $$\underset{A\to \alpha ,\alpha \in {\left(\Sigma \cup N\right)}^{*}}{\sum }\mathrm{Pr}(A\to \alpha )=1.$$

A probabilistic grammar is *consistent* if a sum of probabilities of generation for all strings belonging to a given language is equal to one:

(7) $$\underset{x\in \Sigma^{*}}{\sum }\mathrm{Pr}(S\overset{*}{\Rightarrow }x)=1,$$

where $S\overset{*}{\Rightarrow }x$ denotes all possible derivations starting from *S* and resulting in a finite string *x*. In other words, all the probability mass of the grammar is used for the finite strings it derives [105]

A parser algorithm can be used to calculate the probability *P*(*x*|*G*) that a given sequence *x* was generated by a certain grammar *G* (i.e. the sequence *x* belongs to the language *L*(*G*)) and find the single most likely derivation for *x*. The probability *P*(*x*|*G*) is defined as the product of probabilities of all grammar rules involved in the construction of the corresponding parse tree. Parsing of PCFG is efficient since its computational complexity is polynomial (less than *O*(*n*^3^)) regarding the string length [104,106,107].

Grammar parameters inference by maximum likelihood consists on assigning rule probabilities to maximize the probability *P*(*X*^′^|*G*^′^) over a training set of strings *X*^′^. Given a complete fixed data *X*, *P*(*X*|*G*^′^) is a likelihood function of grammatical model *G*^′^, i.e. the higher the likelihood, the more *G*^′^ fits the data. Closeness of a model to the data can be measured by relative enthropy (also called *Kullback‐Leibler distance*) between two distributions *p*=*P*(*X*|*G*) and *q*=*P*(*X*|*G*^′^) [104]:

(8) $$\begin{array}{l} D(p∥q)=-\underset{x}{\sum }p(x)\cdot \log\frac{q(x)}{p(x)}, \end{array}$$

(9) $$\begin{array}{l} D(p∥q)=-\underset{x}{\sum }p(x)\cdot \log\;q(x)+\underset{x}{\sum }p(x)\cdot \log\;p(x). \end{array}$$

In case of model optimization, the term $\underset{x}{\sum }p(x)\cdot \log\;p(x)$ remains constant, therefore minimization of *D*(*p*∥*q*) is equivalent to minimization of the cross‐enthropy *H*_*p*_(*q*), which is an expected value of -*log*(*q*) under the true distribution *p*:

(10) $$H_{p}(q)=-\underset{x}{\sum }p(x)\cdot \log\;q(x),$$

It can be estimated by averaging over the fixed training sample *X*^′^[104]:

(11) $$H_{p}(q)\approx -\frac{1}{\left|X^{\prime }\right|}\underset{x\in X^{\prime }}{\sum }\log\;q(x)),$$

where |*X*^′^| is a size of the training sample *X*^′^. The RHS expression of Eq. 11 is proportional to the logarithm of the likelihood by a factor of $-\frac{1}{\left|X^{\prime }\right|}$[104].

Every proper PCFG is consistent if it minimizes the cross‐entropy function between probability distributions of the sample and the model [105]. Since a consistent grammar distributes a unit probability over a spectrum of possible sequences, maximization of probabilities of the positive sample is always coupled with minimization of probabilities of the negative sample. Consequently, unlike non‐probabilistic grammars, a probabilistic grammar does not require a negative sample for successful induction.

Practically, two main approaches are used for learning probabilities of grammar rules: Expectation‐Maximization algorithms (EM) [12,13,15,108] and evolutionary methods (e.g. Genetic Algorithms (GA) [37,42,109]‐ [115] or Genetic Programming (GP) [116,117]). A standard EM‐based approach using the Inside‐Outside algorithm [118] is highly sensitive to the choice of initial parameters and usually results in complex grammars, which are difficult to interpret [113]. Instead, we chose a GA‐based approach, which allows customizing the objective function and genomic operators in order to facilitate escaping local optima (see Inference rule weights below).

### Probabilistic model of protein languages

**Figure 1:**
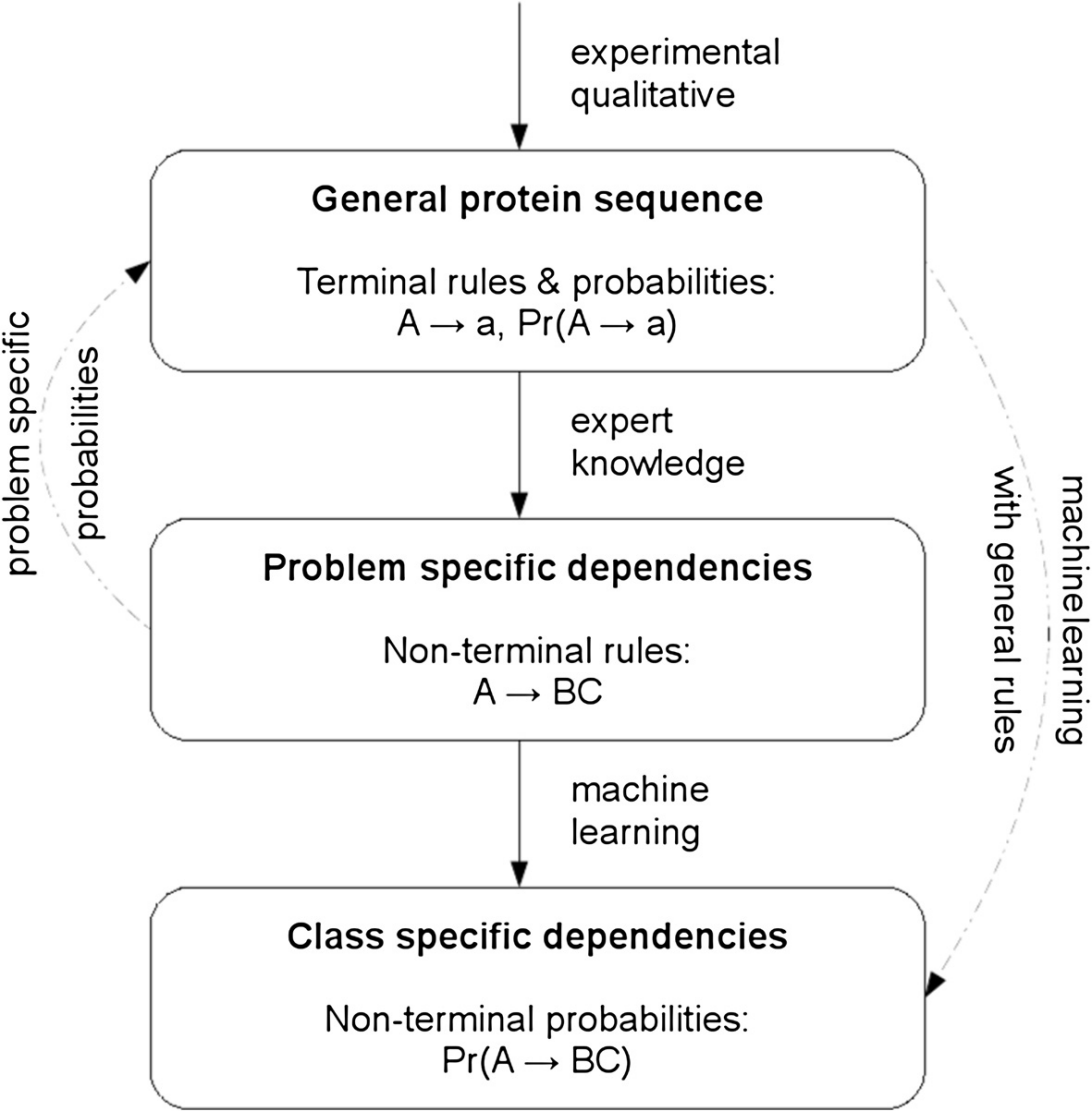
3‐Layer probabilistic grammatical model of protein languages.

We present an original probabilistic grammatical model of protein languages (see [1,9,119] for use of the term). The model covers the lexical (primary structure) and syntactical (secondary and tertiary structure) levels of protein linguistics. Our probabilistic grammatical model can be divided into 3 layers, reflecting steps in the associated grammar induction framework (Figure 1). The first layer is a probabilistic model of a general protein sequence. At this level, experimental quantitative properties of amino acids are assigned to their identities using a fuzzy mapping. In the second layer, a generic set of grammar rules is provided. Preferably, the rules are constrained based on expert knowledge of general dependencies between residues in protein sequences of interest [37,42]. The main purpose of this step is to limit the GA search space in the next layer. In principle, this step could be omitted and an unconstrained grammar could be passed to the next step [37,120]. The final layer narrows the grammatical description down to certain classes of proteins, which are defined in terms of rule probabilities assigned by a machine learning method.

#### Probabilistic model of general protein sequences

Since a protein is generally defined by a string composed of 20 different characters (amino acids), a protein grammar is expected to rely on a large set of terminals. We proposed to utilize physico‐chemical properties of amino acids, collected from the Amino Acid index database (AAindex) [121]‐ [124], to reduce significantly the number of possible combinations of the Right‐Hand Side symbols in production rules [37,115]. Relations expressed by these rules refer to 3 levels of a quantitative property instead of 20 amino acid types, e.g. small, medium or large van der Waals volume. In this way, a number of rules, which is maintainable in the learning process, is kept.

For each given property, our method relies on defining all the rules of the form *A*→*a* (called terminal rules) and associating them with *proper* probabilities, which are calculated using the known quantitative values *pval* associated with the amino acid identities. Three non‐terminal symbols (*Low*, *Medium* and *High*) are created to represent *low*, *medium* and *high* level of the property of interest:

(12) $$\begin{array}{l} \forall a_{i}\;\mathrm{low}\to a_{i},\mathrm{Pr}(\mathrm{low}\to a_{i})\\ \forall a_{i}\;\text{Medium}\to a_{i},\mathrm{Pr}(\text{Medium}\to a_{i})\\ \forall a_{i}\;\text{High}\to a_{i},\mathrm{Pr}(\text{High}\to a_{i}) \end{array},$$

where *a*_*i*_ is the *i*th amino acid type. These non‐terminals are later called *property non‐terminals* (pNT). Symbol *Single* will be used to refer to a pNT unspecifically:

(13) $$\text{Single}\in \left\{\mathrm{low},\text{Medium},\text{High}\right\}.$$

Each amino acid identity *a*_*i*_ is associated with *Single* non‐terminals with a probability Pr (*Single*→*a*_*i*_). For a given property, probabilities are calculated using the known quantitative values *pval*. First, each *pval* is scaled to *pval*^′′^∈[-1,1]. Our original scaling procedure from *pval* to *pval*^′′^[37,115] has been refined in order to account for the residue composition bias found in helices. This *problem‐based scaling* scheme is defined as follows:

(14) $$\begin{array}{l} \mathrm{pva}l^{\prime }(a_{i})=\mathrm{pval}(a_{i})-\underset{j}{\sum }\mathrm{pval}(a_{j})\cdot \mathrm{prop}(a_{j}),\\ \mathrm{pva}l_{L}^{\prime \prime }(a_{i})=\frac{\mathrm{pva}l^{\prime }(a_{i})}{\mathrm{mi}n_{j}\mathrm{pva}l^{\prime }(a_{j})},\\ \mathrm{pva}l_{H}^{\prime \prime }(a_{i})=\frac{\mathrm{pva}l^{\prime }(a_{i})}{\text{ma}x_{j}\mathrm{pva}l^{\prime }(a_{j})}, \end{array}$$

where *prop*(*a*_*j*_) is the proportion of amino acid *a*_*j*_ in the problem of interest. In practice, *prop*(*a*_*j*_) are estimated from amino acid composition of combined positive and negative learning sets.

The main rationale behind this scheme is that an average value of property in the environment of interest is often very specific. For instance, *alpha*‐helices consist of small and accessible residues (see Additional file 1: Figure S1). This bias should be taken into account in the process of design of terminal rules in order to use property space efficiently.

Terminal rule probabilities are calculated for each amino acid type *a*_*i*_, using the following formulæ:

(15) $$\begin{array}{c} \mathrm{Pr}(\mathrm{low}\to a_{i})=\left\{\begin{array}{cc} \frac{\mathrm{pva}l_{L}^{\prime \prime }(a_{i})}{\underset{j}{\sum }\mathrm{pva}l_{L}^{\prime \prime }(a_{j}),} & \;\mathrm{if}\mathrm{pva}l_{L}^{\prime }<0\\ 0 & \;\mathrm{if}\mathrm{pva}l_{L}^{\prime }\geq 0 \end{array}\right)\\ \mathrm{Pr}(\text{High}\to a_{i})=\left\{\begin{array}{cc} \frac{\mathrm{pva}l_{H}^{\prime \prime }(a_{i})}{\underset{j}{\sum }\mathrm{pva}l_{H}^{\prime \prime }(a_{j}),} & \;\mathrm{if}\mathrm{pva}l_{H}^{\prime }>0\\ 0 & \;\mathrm{if}\mathrm{pva}l_{H}^{\prime }\leq 0 \end{array}\right)\\ \mathrm{Pr}(\text{Medium}\to a_{i})=1-(\mathrm{Pr}(\mathrm{low}\to a_{i})+\mathrm{Pr}(\text{High}\to a_{i})) \end{array}.$$

Note that these probabilities are proper.

Based on our preliminary study [42] the properties of normalized *van der Waals volume*[125] and information value for accessibility with average fraction of 35% (called later *accessibility*) [126] were chosen as the basis for the grammars. They are representatives of physiochemical properties and hydrophobicity categories in AAindex, respectively [122].

#### Problem‐specific dependencies

The next layer of our probabilistic grammatical model is dedicated to formalize dependencies between amino acids in the form of non‐terminal grammar rules. Whereas one could use a non‐constrained set of rules and attempt to learn their associated probabilities (top‐down arrow in Figure 1), the complexity of the machine learning problem is such that this would not be practical. Therefore, we decided to use a grammatical model specially designed according to expert knowledge to represent helix‐helix contacts [35]. An advantage of problem‐specific rules is that the number of non‐terminal symbols can be increased, while keeping the same number of rules in a grammar, since the number of non‐terminal symbols defines the degree of complexity of the dependencies which could be modeled by a grammar.

Moreover, we found previously [37,115] that, to avoid trivial solutions of the learning, the grammar model should not allow usage of the same LHS NT symbols in the terminal and non‐terminal rules.

#### Grammatical model of helix‐helix contact sites

Since helix‐helix contact site motifs should represent direct dependencies between sequences of two helices, a linear sequence pattern or profile cannot be used as descriptor. A more expressive representation is required. Given that probabilistic context‐free grammars or their associated parse trees are able to represent pairwise nested dependencies between residues from two helices, they appear as suitable descriptor candidates. The major difficulty is, however, that the specific amino acid composition and periodicity of *α*‐helices create a strong helical signal in the sequence, which can dominate a subtle helix‐helix contact signal during learning process. Therefore, the weak contact class‐specific signal needs to be extracted from the strong helical noise, in order to allow modeling of the contact sites. We address this by constraining grammar structures so that they reflect general helix‐helix contact site features, such as the helix periodicity and residue pairing.

Helix interface is defined as a set of residues that are in contact with residues from the other helix. The residues of the inner or contact face (*interface*) of a helix are separated by either 1 or 2 residues of the *outerface* so that an average helix periodicity of 3.6 residues is preserved. Two helices are separated by a string of amino acids (*turn*). The *anti‐parallel* configuration of a helix pair can be described schematically by context‐free grammar rules, such as [35]:

(16) Interface→InsideRes1OuterfaceInsideRes2|Turn

Outerface→OutsideRes1InterfaceOutsideRes2|Turn,

where *InsideRes*1 means one or two residue(s) of the interface of helix 1; | separates alternative right hand side symbols.

Now, we present a grammar *G*, capable of modeling a helix pair, which imposes helix periodicity (3‐4 residues) and keeps the computational cost of inference low enough (e.g. not extending ca. 200 rules [37]). This grammar *G* is a modified version of the non‐probabilistic context‐free grammar *G*_*pair*_ proposed in [35].

The basic alphabet *Σ*_*AA*_ consists of a set of 20 symbols representing 20 standard amino acid identities:

(17) $$a_{i=1\mathrm{..}20}\in \Sigma_{\mathrm{AA}}=\left\{\begin{array}{c} A,R,N,D,C,E,Q,G,H,I\\ L,K,M,F,P,S,T,W,Y,V \end{array},\;\right\}\;.$$

Moreover, an *empty* word *ε* and four additional symbols: [, ], { and } are added, which denote boundaries of helix 1 and helix 2, respectively:

(18) $$\Sigma =\Sigma_{\mathrm{AA}}\cup \left\{\left[,],\{,\right\}\right\}.$$

The set of non‐terminal symbols *N* is defined as follows:

(19) $$N=\left\{\begin{array}{l} \mathrm{low},\text{Medium},\text{High},\\ \text{Doubl}e_{A},\text{Doubl}e_{B},\text{Doubl}e_{C},\\ \text{Interfac}e_{\mathrm{DD}},\text{Interfac}e_{\mathrm{SD}},\text{Interfac}e_{\mathrm{DS}},\text{Interfac}e_{\mathrm{SS}},\\ \text{Outerfac}e_{P},\text{Outerfac}e_{\mathrm{SD}},\text{Outerfac}e_{\mathrm{DS}},\text{Outerfac}e_{\mathrm{SS}},\\ \text{Turn},\text{Whatever},\text{Any},\\ \text{Start} \end{array}\right\}.$$

*Low*, *Medium* and *High* are three property level non‐terminals, as defined in Eq. 12‐ 15. *Double*_*A*_, *Double*_*B*_ and *Double*_*C*_ are three non‐terminals to represent two consecutive amino acids in a helix. They facilitate expression of helix periodicity with a limited number of rules. (Note that, in principle, an arbitrary number of *Double*_*X*_ symbols could be used in order to adjust the expressive capability of the grammar). Symbol *Double* will be used to refer to any of them unspecifically:

(20) $$\text{Double}\in \left\{\text{Doubl}e_{A},\text{Doubl}e_{B},\text{Doubl}e_{C}\right\}.$$

Four *Outerface* and four *Interface* NT symbols are in the core of the model. Subscripts *DD*, *SD*, *DS*, *SS* denote *singular* or *double* length of the context of interface or outerface fragments. *Outer*-*Interface* rules ensure that each complete helix turn is 3 or 4 amino acids long. For example, *Outerface*_*SS*_, which is surrounded by two single residues, can only be followed in the derivation by *Interface*_*DD*_ which is surrounded by two double residues (see Eq. 22). The non‐terminal *Turn* covers the part of a protein sequence which is between fragments of helices in contact. *Whatever* refers to a random string of amino acids. It increases flexibility of the grammar and could account for weakly constrained residues in the ends of the contact region. *Any* non‐terminal means any amino acid. Finally, *Start* is the starting symbol of the grammar.

The set of rules *P* is defined in the following way:

(21) $$\begin{array}{c} \forall a_{i}\;\begin{array}{l} \mathrm{low}\to a_{i}\\ \text{Medium}\to a_{i}\\ \text{High}\to a_{i}\\ \text{Any}\to a_{i} \end{array} \end{array},$$

It is important to note that, strictly speaking, the grammar structure, described above, allows representing dependencies between types of residues from two helices rather than actual residue‐residue contacts. However, it is a reasonable assumption that spatial residue‐residue contacts are likely to imply some constraints on residues and thus define the dependencies. However, there is no implication that a dependency indicates a spatial contact.

#### Inference of rule weights

A positive training set containing examples of sequences of the protein structure of interest was used to infer rule weights. A single individual in GA represented the rule probabilities of a whole grammar. The general principle was to start the learning process with the complete set of rules expressing prior general knowledge of the protein domain. Although this approach leads to quite large sets of rules (approximately 200) even for moderate alphabets, it avoids bias which would be introduced by additional constraints. Goodness of each individual in the population was evaluated by parsing the entire positive training set in every epoch of GA. During training, rule probabilities were inferred to express class‐specific dependencies. Only one best grammar in a single run of GA was recorded. As convergence of the genetic algorithm to the global optimum cannot be guaranteed, for each grammar generation setup, we typically performed several runs.

The genotype consisted of a single chromosome coded with a string of real numbers (<0,1>) linked to grammar rule probabilities. Gene values were normalized in order to obtain *proper* probabilities. Only probabilities of non‐terminal rules were evolved. The original population of 200 individuals, representing grammars, was initialized randomly and then iteratively subjected to evaluation, selection, reproduction and genomic operators [37]. A non‐linear genotype to phenotype function was used to facilitate rapid convergence and enhance exploring capabilities of the genetic algorithm [42].

The objective function of the GA (the function to be maximized) was defined as an arithmetic average of logarithms of probabilities returned by the parsing algorithm for all positive training samples. In our framework, an implementation of the Earley algorithm for probabilistic parsing [103,104,107], where a probability for a certain node is calculated as a sum of probabilities of all sub trees, was used for training during grammar induction. The arithmetic average of logarithms of a sum of probabilities of all sub trees, given by the parser, is an estimator of cross‐enthropy between the model and sample distributions (Eq. 11), as discussed in [104]. Therefore, the grammar induction in our framework was a cross‐entropy minimization. Since all evolved grammars were proper, and, therefore, consistent [105], negative samples were not necessary for successful induction.

The fitness score (the measure of goodness of an individual) was calculated from the objective function by accomodating the diversity pressure. This improves exploratory abilities of the GA ‐ settling in local extremes of the fitness landscape is less likely [109,110]. This was done by using a triangular sharing function that decreased fitness score of individuals on the basis of their similarity to other individuals in the population [110,127]. The cutoff value of the sharing function, initially 10.0, was multiplied by 2 adaptatively when improvement of fitness scores slowed down. This shifted a focus from exploration of entire space towards inspection of local optima neighborhoods in the late stages of the evolution. The similarity between chromosomes was measured using distance *Dist*_*WH*_ introduced in [37].

The reproduction step relied on the tournament method with 2 competitors [37,42,128]. A steady‐state GA with 50% overlap was used. Offsprings were produced by averaging genetic information of two individuals with a random distortion in order to enhance exploratory capabilities of the algorithm. Subsequently, a classical one point mutation operator was used to mutate randomly chosen genes. The probabilities of crossover and mutation were 0.9 and 0.01, respectively. We found that the simultanous use of 50% overlap between populations and the random distortion ensured stable evolution of a reasonably diverse population. Subsequent tests of an alternative approach, utilizing elitism, showed deteriorated performance. Indeed, the setup of our GA represented the optimum achieved in empirical tests.

The algorithm stopped when there was no further significant improvement in the best scores (ratio 1.001 over 100 iterations). After grammar induction, the fraction of rules with near‐zero probability is typically high, because assigning non‐zero probabilities to rules which are never used in any derivation would decrease grammar fitness to the sample (note that the grammar is *proper*). The final set of rules could be pruned from these low probability rules, which have a limited impact on the overall score of a scanned sequence. Therefore, our inference of rule weights could be seen also as a selection of rules from a generic set.

The implementation of our grammar induction algorithm was based on M. Wall’s GAlib library, which provides a set of C++ genetic algorithm objects [127].

#### Scanning of protein sequence

Viterbi style Earley algorithm was used for scanning, where a probability for any node in the parse tree was calculated as a maximal probability from all sub trees. Not only the Viterbi algorithm produces good discrimination between positive and negative samples, but the most likely parse tree may reflect structural features of a molecule [12,37].

The aim of this work is a sequence‐based classification of transmembrane helix‐helix contact sites within 4 main structural classes [91]. Two approaches to the classification problem are tested. In the first case, a grammar descriptor is trained for each class. Then, it assigns a score that reflects probability of belonging to the class to every helix pair sequence in the test set. To increase robustness of the classification to local minima of the inference process, scores obtained for the same sequence from several grammars trained for the same class could be averaged in order to produce a *combined grammar classifier*.

In the second approach, grammar descriptors trained for different classes are combined into a *multiple grammar classifier*, as described in detail in the next section. In this case, the classification problem is treated as a 4‐way classification.

#### Multiple grammar classifier

*Multiple grammar classifier* was designed for 4‐way classification problem by combining predictions from grammars trained separately for the four contact classes. The rational behind this scheme is that a sequence *w*_*i*_ of class *q* is expected to obtain a high score from the grammar trained for this class and low scores from grammars trained for other 3 classes. The new score returned by the multiple grammar classifier, called *multiscore*, is defined by the following equation:

(23) $$\text{multiscor}e_{q}(w_{i})=\log{\mathrm{Pr}}_{q}(w_{i})-\frac{1}{3}\underset{{}_{p=1\mathrm{..}4,p\neq q}}{\sum }\log{\mathrm{Pr}}_{p}(w_{i}),$$

where *w*_*i*_ is a sequence of interest, and *q* is a contact site class. *log*Pr_*q*_(*w*_*i*_) is the log probability of a sequence *w*_*i*_ obtained by the Viterbi style parser using a grammar trained for class *q*.

#### Averaged parse tree

We propose a novel averaged *a priori* parse tree representation which conveys additional information allowing the analysis of grammar descriptors. First, one may observe that each parse tree of a grammar in the form expressed by Eq. 22 has exactly one stem defined by the interleaving symbols *Interface*/*Outerface* of different types. The types, denoted by the subscripts *DD*|*SD*|*DS*|*SS*, determine *whether theInterface*/*Outerfaceis surrounded by one or two residues on each side (each side represents*a helix). Therefore, given a sequence of *Interface*/*Outerface* symbols used in the derivation between *Start* and *Turn*, the number of residues originating from each helix at each step of the derivation is known, e.g. *Start*⇒*Outerface*_*DD*_⇒*DoubleInterface*_*DS*_*Single*⇒*SingleOuterface*_*SD*_*Double*⇒*Turn*.

Now, let us assign numerical values to property level non‐terminals (see Eq. 21), such that:

(24) val(low)=0,val(Medium)=1,val(High)=2.

Then, for every non‐terminal *Double*, average level of property at position 1 and 2 can be calculated according to grammar rule probabilities. The values would reflect the preferred level of property, in the range of <0,2>, at a given position in the derivation starting with a certain *Double* symbol.

E.g. let us consider the surrounding of *Interface*_*DS*_ derived from *Outerface*_*DD*_ providing the subset of rules and their probabilities given in Eq. 25.

The right hand site context of *Interface*_*DS*_ is a single residue, whose level is expected to be on average 0×0.5+1×(0.3+0.2)=0.5. In the left hand site context, there are two residues. The average *a priori* property level of the first residue can be calculated as follows: (0.5+0.3)×(0×0.6+1×0.4)+0.2×(1×1.0)=0.52. Similarly, the second residue is expected to have an average level of (0.5+0.3)×(0×0.1+1×0.9)+0.2×(1×0.4+2×0.6)=1.04.

The procedure could be extended for the rules that elongate helix‐helix interfaceto generate a unique average *a priori* parse tree. In the paper, the preferred property levels of the average *a priori* parse tree are represented by the branch lengths (rounded to the nearest integer).

#### Measures of performance

The performance of grammar classifiers was evaluated using the Leave‐One‐Out and 4‐fold Cross‐Validation schemes (LOOCV and 4‐fold CV, respectively) and on an independent test set. The LOOCV procedure consisted of training on all but one positive samples, and then testing efficiency of recognition of that one sample among all negative samples. Similarly, the 4‐fold CV consisted of training on 3/4 of positive samples, and then testing efficiency of classification on the remaining 1/4 of positive samples and all negative samples.

To measure quality of grammars, we use the ROC curves [129,130], defined in terms of *FalsePositive* (FP) and *TruePositive* (TP) rates. *TPrate* is also called *Sensitivity* or *Recall*, while *FPrate* equals to 1-*Specificity*. The measures have an advantage of being independent of relative sizes of positive and negative samples. The *area under ROC curve* or *AUCROC* is used for general assessment of classifier quality and selection of the best grammar. The cumulative character of *AUCROC* parameter makes it convenient for evaluation of the performance of automatically induced classifiers. *AUCROC* is a good indicator of a learning method efficiency [129,130]. In this paper, we always report the average of *AUCROC*s calculated from ROC curves obtained for different cross‐validation subsets.

Note that in the case of LOOCV, the value of *AUCROC* is equal to *Specificity* (or *TrueNegative* rate) at the threshold value equal to the score of the only positive sample. Indeed, at this point, since the *Sensitivity* (or TP rate) increases from 0 to 1, the ROC curve climbs up (ROC curve in the LOOCV can be written as a Heaviside function: *H* [1-*Specificity*(*score*_*thepositivesample*_)]). In other words, the value of *AUCROC* is equal to the fraction of the negative set which obtained worse score than the positive helix pair.

In addition, the ROC curves produced using the vertical averaging method [130] for band length 0.1 are provided for LOOCV and 4‐fold CV. In the case of LOOCV, approximate confidence bars based on normal approximation intervals [131,132] are shown (see [132,133] for critical assessment of the method). Based on the averaged ROC curves, *Precision*, *Recall*, their harmonic mean *F*1, and *Accuracy* are provided for selected *FPrate* thresholds.

For the multiple grammar classifiers, a ROC curve is computed using *multiscore*s.

## Datasets

### Overview

The **Walters and DeGrado dataset** of transmembrane helix‐helix contacts [91], referred as ***WDG***, contains 445 interacting transmembrane helix‐helix pairs whose length varies from 10 to 14 residues per helix. In this study, we only considered the 4 most populous (67% of all cases) types of contact sites out of 14 created by the authors. Those 4 classes, that are referred as *c1‐c4*, contain 300 pairs (130, 71, 57, 42 in classes 1‐4, respectively). The other classes were omitted because they do not include sufficient number of members for automated training of classifiers.

We define a helix pair as *anti‐parallel* if residues in the N‐terminus of the first helix interact with residues in the C‐terminus of the other helix. Analogically, if residues in the N‐terminus of the first helix interact with residues in the N‐terminus of the other helix, we call such a helix pair *parallel*. In the most populous **class 1** (29% of the helix pairs in the *WDG* set), the helices are arranged antiparallelly. The crossing angle is relatively small (24 °±10), while the average geometrical distance between two helices is 8.6 ±0.9 Å. The distinctive feature of the class is a heptad repeat of small residues (glycine (G), alanine (A), serine (S)). Small amino acids are also frequent in the middle of the heptad. **Class 2**, which covers 16% of the helix pairs, is another example of antiparallel packing. While the average geometrical distance between two helices is similar to the one found in class 1 (8.6 ±1.0 Å), the crossing angle is on average greater than in class 1 (34 °±14). At the sequence level, the small residues spaced at four‐residue intervals (i.e. GxxxG motif) form a flat surface approached by larger residues in the opposite helix, spaced at the same interval. **Class 3** (13% of the dataset) is the parallel version of class 2. It has the largest average crossing angle (38 °±8), while the average geometrical distance is the shortest (7.9 ±0.9 Å) among the four classes. Similarily to class 2, GxxxG motif is typically present on one of the two helices. Finally, **class 4**, which accounts for 9% of the helix pairs, has a parallel configuration resembling class 1. Reversely to class 3, it has the smallest average crossing angle (14 °±17) and the largest average geometrical distance (9.8 ±1.2 Å). Interestingly, no position specific propensities for small residues were found in this class by [91].

The number of transmembrane protein structures in the PDB database [44] has increased significantly since mid 2006, when [91] research was completed. Therefore, we created a new **PDBTM dataset** based on the PDBTM database [47]. To avoid closely homologous structures, only 285 non‐redundant (at 40% identity) alpha‐helical chains were extracted from the PDBTM database (as of 30th November 2009). Note that contacts between helices from two chains in homo‐oligomeric proteins are not represented in this dataset. Helix pairs with at least 1 residue in contact, according to Promotif3 [134] definition, were searched. This procedure resulted in the dataset, referred as ***PDBTM***, containing 641 whole helices involved in contacts.

### Construction of datasets

For each of the four main classes c1‐c4, the helix‐helix pair acting as centroid (3D template) in the WDG dataset was selected as class representative. Since processing helix pair sequences of varying lengths introduces an extra level of complexity, which may lead to a decrease in performance, we first truncated those templates to the length of 10‐10 residues using a criterion of the most concise geometrical representation (least sum of inter‐helical spatial distances between C *α* atoms), see Additional file 2: Figure S2. Then we aligned all *WDG* and *PDBTM* pairs to the *WDG* class centroids and the best match over 10‐10 residue length was calculated according to RMSD. Then the fragments were assigned to the four classes according to a RMSD cutoff threshold of 1.50 Å, as in [91], in order to keep only the best pairs. While helical pairs in the *PDBTM* datasetwere assigned to Walters and DeGrado classes, the acceptance rate of helix pairs to a given WDG contact site class, in function of the RMSD cutoff, appeared to be almost linear for the range of thresholds from 1 to 2.5 Å(see Additional file 3: Figure S3). We surmise that the conformation space of transmembrane helix‐helix contact sites may be continuous with classes c1 and c2, respectively c3 and c4, merging into a unique anti‐parallel, respectively parallel, class. Therefore, the WDG classes could be seen as a way of quantitizing the space of helical pair configurations.

This resulted in two datasets: 

• ***WDG150NR***: A set of 140 helix pair fragments (67, 35, 23 and 15 in classes 1‐4, respectively) based on the Walters and DeGrado dataset. The set represents 31% of all helix pairs in the original Walters and DeGrado dataset (47% of pairs from classes 1‐4).

• ***PDBTM150NR***: A set of 227 helix pair fragments (100, 60, 25 and 42 in classes 1‐4, respectively) based on the PDBTM dataset. The set represents 35% of all helix contact sites and 45% of helix contact sites involving at least 3 residues found in the PDBTM dataset.

The sets were made not‐redundant and thus mutually independent by applying the Decrease redundancy application (http://web.expasy.org/decrease\_redundancy/) with 40% maximum identity level. A lower similarity threshold would significantly reduce the number of sequences, e.g. to less than 50 in *the entireWDG150NR* in the case of 20% identity. Moreover, any identity threshold below 50% could result in an undesirable exclusion of helix pairs, in which one helix is involved in another pairing in the same class. However, at the 40% identity level, some extent of intra‐class weak homology is likely. In order to evaluate this, we counted the number of sequences in *WDG150NR* having at least one intra‐class or inter‐class neighbor whose identity was between 20% and 40%. We found that 70% of sequences have an intra‐class neighbor, while 86% have an inter‐class neighbor in that identity range. This shows that sequence identity with the 20‐40% range does not implicate contact class membership, although we observed that shared sequence identity increased grammar performance (see Analysis and discussion).

The datasets are summarized in Table 1. Note that percentages of helix‐helix contact sites from *WDG* and *PDBTM* datasets assigned to the four classes were similar for the most populous classes 1 and 2. Class 4 was overrepresented in the *PDBTM* at the expense of class 3 (see Additional file 2: Figure S2).

**Table 1:** Datasets summary

**Dataset/Class**	**Centroid (PDB)**	**Size**	**Fraction**
**WDG150NR**		**140**	**100%**
c1	1Q90 B119‐128 B194‐203	67	48%
c2	1OKC A114‐123 A180‐189	35	25%
c3	1J4N A18‐27 A101‐110	23	16%
c4	1RH5 A29‐38 C34‐42	15	11%
**PDBTM150NR**		**227**	**100%**
c1	1Q90 B119‐128 B194‐203	100	44%
c2	1OKC A114‐123 A180‐189	60	26%
c3	1J4N A18‐27 A101‐110	25	11%
c4	1RH5 A29‐38 C34‐42	42	19%

## Results

### Cross‐validation of grammar classifiers

#### Leave‐one‐out cross‐validation

Performance of single grammar classifiers was analysed in the Leave‐One‐Out Cross‐Validation scheme with the *WDG150NR* dataset. The correctness of the leave‐one‐out procedure was assured by the non‐redundant design of the set. Only one grammar was trained for each subset and amino acid property using all but one positive sample of a given class of helix pairs. The performance of the classifiers was tested on the one remaining positive sequence from that class and all negative sequences from the 3 other classes (class‐vs‐3 classification). The classifiers were evaluated on the basis of their average *AUCROC* which in the case of LOOCV is equal to *Specificity* at the threshold equal to the score of the only positive sample (Table 2).

**Table 2:** Leave‐one‐out cross‐validation

**Trained for**	**Accessibility**	**vdW volume**
c1	**0.54 ±0.04**	0.41
c2	0.46	**0.70 ±0.04**
c3	0.50	**0.56 ±0.07**
c4	**0.58 ±0.09**	0.33

Average *AUC ROC* of class‐vs‐3 classification of helix‐helix contact fragments *in theWDG150NR* dataset. Standard deviation of the mean is given for *AUC ROC* greater than 0.50.

The best average *AUCROC* in the *WDG150NR* ranged from 0.54 ±0.04 for accessibility‐based grammar of class 1 to 0.70 ±0.04 for van der Waals‐based grammar of class 2. The highest *AUCROC* values were achieved by van der Waals volume based grammars for classes 3 and 2 whereas the best results for classes 1 and 4 were obtained using accessibility based grammars (Table 2). Besides the best performing grammars, classifiers trained for properties, which were *not* the most informative for a given class, e.g. van der Waals volume for classes 1 and 4, and accessibility for classes 2 and 3 obtained average *AUCROC* below 0.50.

ROC curves (with approximate 95% confidence intervals) produced using the vertical averaging method are provided in the Additional file 4: Figure S4. *Precision*, *Recall*, *F*1 and *Accuracy* are provided at selected FP rates in the Additional file 5: Figure S5.

Interestingly, sequences left out such that subclasses of *AUCROC* equal or higher than 0.75 could be distinguished (Additional file 6: Table S1). For instance, for van der Waals‐based grammar of class 2, the high specificity subclass consisted of 51% of samples. The average *AUCROC* in the subclass was 0.89 in comparison to 0.70 in the whole class 2. Most notably, for accessibility‐based grammar of class 4, 47% of samples (7 out of 15) where classified with *AUCROC* equal or higher than 0.75. The average *AUCROC* in this subclass reached 0.90 in comparison to just 0.58 in the whole class 4. A statistically significant correlation between AUC ROCs and average sequence alignment distances (see Analysis and discussion) suggests that the high specificity subclasses originate from the presence of closer neighbors in the training set.

#### Four‐fold cross‐validation

**Figure 2:**
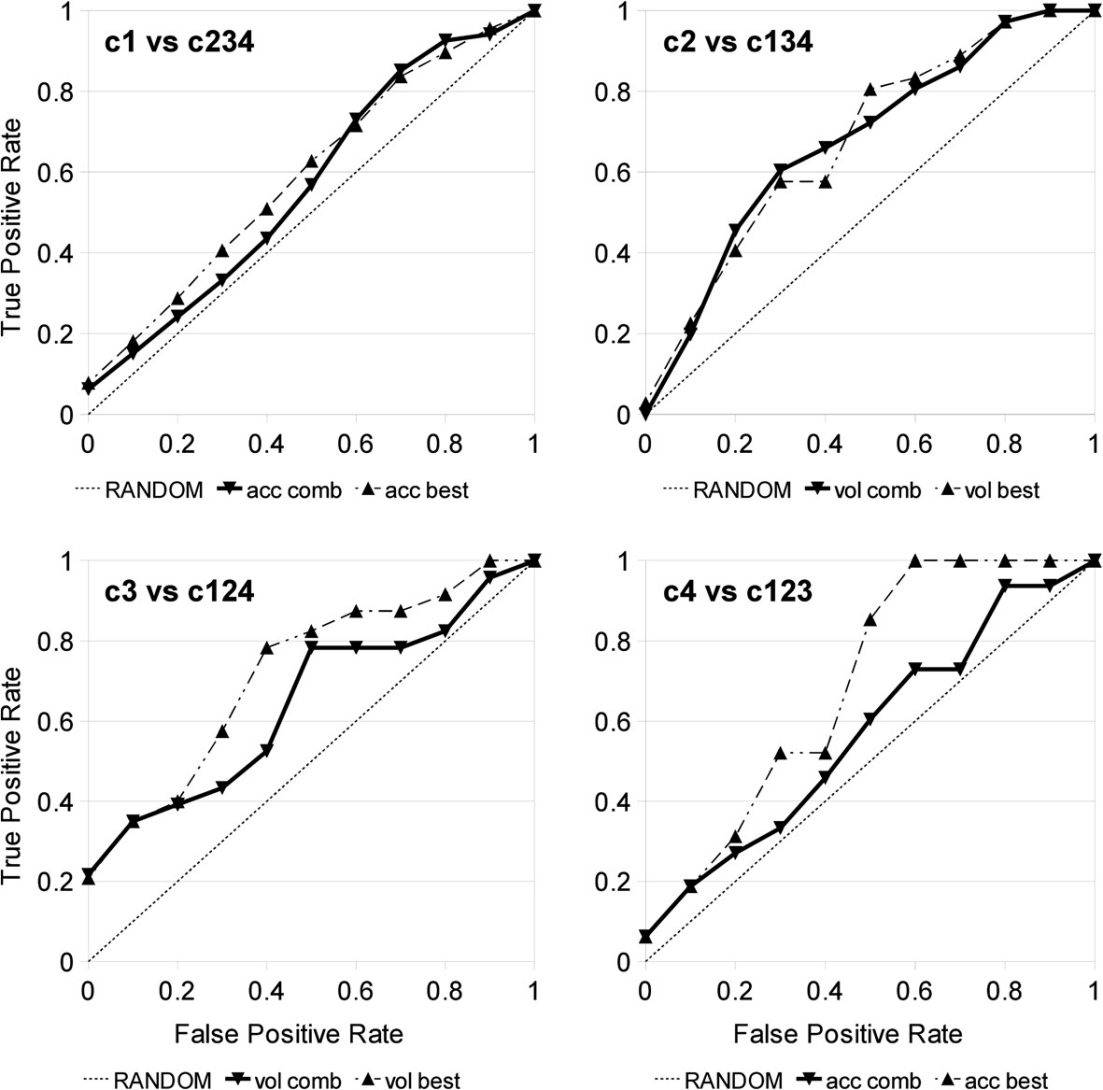
**ROC Curves in the Four‐fold Cross‐Validation.** ROC curves of combined (solid line) and best (dashed line) grammars in 4‐fold CV of helix‐helix contact fragments classification in *WDG150NR*.The ROC curves were produced using the vertical averaging method for band length 0.1. Notation: *acc* ‐ accessibility Aaindex:BIOV880101;*vol* ‐ van der Waals volume Aaindex:FAUJ880103).

(4‐fold CV) was carried out on the same dataset. The division was made according to alphabetical order of helix pair identifiers derived from the PDB codes. For each of the 4 helix‐helix contact site classes and 2 selected amino acid properties (8 cases in total), 3 grammars were generated using 3/4 of positive samples (Note that, despite using the same set of parameters, GA usually produces different solutions in each run). Moreover, *combined* grammar classifiers were created by averaging scores (log probabilities) assigned to helix pairs by 3 grammars trained for the same class and amino acid property. Performance of the single and combined classifiers was tested on the remaining 1/4 positive sequences and all negative sequences in the dataset. The predictive power was assessed in class‐vs‐3‐other‐classes classification. Then, *AUCROC* scores achieved by the combined classifiers were averaged over the 4 subsets of the *WDG150NR*. In addition, the best single grammars, in terms of *AUCROC*, were selected out of the 3 grammars per each class‐property‐subset case. Their *AUCROC* scores, also averaged over the 4 subsets, represent potential performance of the method (see the Limitations section of the Analysis and discussion). The averaged classification performance of the combined grammars and best single grammars is shown in Table 3. ROC curves produced using the vertical averaging method are shown in Figure 2. *Precision*, *Recall*, *F*1 and *Accuracy* are provided at selected FP rates in the Additional file 7: Table S2.

**Table 3:** Four‐fold cross‐validation

**Trained for**	**Using property**	
	**Accessibility**	**vdW volume**
c1	**0.55 (0.58)**	0.41 (0.46)
c2	0.46 (0.50)	**0.68 (0.69)**
c3	0.50 (0.55)	**0.61 (0.71)**
c4	**0.57 (0.71)**	0.44 (0.55)

Average AUC ROC of combined grammars in 4‐fold CV of helix‐helix contact fragments classification in *WDG150NR*. Average AUC ROC of the best single grammars out of 3 trained grammars for each class‐property case is shown in parentheses. Bold fonts indicate the best performance for a given helix contact site class.

The performance of combined grammars trained for the amino acid property most useful for a given class (bold in Table 3) varied from 0.55 to 0.68 (average 0.60) in terms of *AUCROC*. These values are similar to *AUCROC*s in the LOOCV. The average *AUCROC*s of best single grammars for given class (bold in parentheses in Table 3) varied from 0.58 to 0.71. Significantly, the best single grammars were 14‐25% better than combined grammars for classes 3‐4. Notably, van der Waals volume based grammars for class 3 recognized over 20% of positive samples without an error (Figure 2). Eventually, selecting the best grammars and most appropriate properties, the average *AUCROC* over 4 classes was 0.67 (average weighted by relative numbers of samples in each class was 0.64).

#### Multiple grammar classifiers

The helix pair classification problem can be treated explicitely as a 4‐way classification problem. For this purpose, multiple grammar classifiers were build on the basis of the best combined grammars trained in the 4‐fold Cross Validation procedure (c1, c4 ‐ accessibility grammars, c2, c3 ‐ van der Waals volume grammars). The performance of the multiple grammars classifiers and the combined grammar is compared in Table 4. For a given contact site class *q*, 3/4 of its positive and negative sets were used to calculate *multiscore* (Eq. 23). Thus only the remaining 1/4 of the positive and negative sets were used for testing in the multiple grammar classifier scheme. Therefore, for performance comparison, *AUCROC*s of the combined grammars were recalculated using only this remaining 1/4 of the negative set. The lower cardinality of the negative sample did not affect *AUCROC*s of the combined grammars by more than 0.03.

**Table 4:** Multiple grammar classifiers

**Class**	**WDG150NR**		**PDBTM150NR**	
	**Combined**	**Multi**	**Combined**	**Multi**
c1	0.56 (0.59)	0.66	0.47 (0.48)	0.50
c2	0.67 (0.68)	0.62	0.54 (0.53)	0.54
c3	0.64 (0.71)	0.65	0.60 (0.59)	0.57
c4	0.57 (0.70)	0.66	0.57 (0.55)	0.61
avg	0.60	0.65	0.52	0.54

Average AUC ROC of the combined and multiple grammar classifiers in *WDG150NR* (4‐CV) and *PDBTM150NR* datasets. All results refer to grammars trained using *WDG150NR* in the 4‐CV experiment. Test performance of the single grammars, which were the best in 4‐fold CV in *WDG150NR,* is shown in parentheses. *avg* is an average weighted by relative numbers of samples in each class.

The average *AUCROC* increased from 0.60 to 0.65 for the multiple grammar classifiers in comparison to the combined grammars. The largest improvement (up to 16‐17%) was obtained for classes 1 and 4. Only performance of class 2 grammars recognition decreased by 8%. All multiple grammar classifiers obtained *AUCROC* above 0.6. The performance of the multiple grammars classifiers was similar for all the classes, as this type of classifier utilized scores generated by combined grammars trained for each class. Overall, the multiple grammars classifiers benefited from combining positive and negative information.

### Test on independent dataset

Grammars infered in 4‐fold CV using the *WDG150NR* dataset were applied to classification of helix‐helix contact fragments in the *PDBTM150NR*dataset, created by us independently (Table 1). Multiple grammar classifiers, combined single grammars and best single grammars according to 4‐fold CV were evaluated (Table 4).

The best results were obtained by the combined van der Waals‐based grammar of class 3 (*AUCROC* of 0.60) and the combined accessibility‐based grammar of class 4 (*AUCROC* of 0.57). In these cases performance in the *PDBTM150NR* matched performance in the *WDG150NR*. S*ingle grammars which performed best inWDG150NR* achieved ca. 20% worse results in the *PDBTM150NR*. All grammar classifiers trained for class 1 and 2 performed poorly in the *PDBTM150NR*. There was no significant benefit when the multiple grammar classifiers were applied.

#### Training on PDBTM dataset

Noticeably worse results, when the *PDBTM150NR* was used for testing, could indicate some incoherence between the *WDG150NR* and *PDBTM150NR* datasets. The hypothesis was investigated in the opposite scenario, where the whole *PDBTM150NR* was used for training and the whole *WDG150NR* was used for testing (Table 5, left). If *PDBTM150NR*’s sequence patterns coverage is wider than *WDG150NR*’s, performance similar to 4‐fold CV (Table 3) is expected.

**Table 5:** PDBTM training

**Trained for**	**Using property**	
	**Accessibility**	**vdW volume**
c1	**0.56 (0.58)**	0.44 (0.47)
c2	0.45 (0.49)	**0.60 (0.62)**
c3	0.44 (0.48)	**0.59 (0.65)**
c4	**0.56 (0.63)**	0.32 (0.45)

Average AUC ROC of combined grammars trained in *PDBTM150NR* in helix‐helix contact fragments classification in *WDG150NR*. Average AUC ROC of the best performing single grammars out of 3 trained grammars for each class‐property case is shown in parentheses. Bold fonts indicate the best performance for a given helix contact site class.

Indeed, the classifiers obtained in this way showed better performance in the *WDG150NR* than combined grammars trained in the *WDG150NR* and tested in the *PDBTM150NR* datasets for classes 1 and 2 (10‐20%, Table 4). Performance of grammars for classes 3 and 4 was similiar. In general, these results support the hypothesis that *PDBTM150NR*, while coherent with *WDG150NR* in terms of spatial similarity (RMSD), covers a wider space of sequence patterns.

### Comparison with standard methods

In this section, we compare our PCFG‐based method with other approaches, namely simple BLAST search, closest neighbor search in the training set, and Profile HMM.

#### Classifiers

BLASTP [135] is a standard tool for protein sequence similarity searches (we use the recent NCBI version 2.2.28+). The idea behind this test was to demonstrate that the helix‐helix contact site classification could not be solved trivially by applying BLASTP to find similar sequences. For each helix‐helix contact site class, its training set was used as a query and respective test set as a subject (or database). Subject sequences belonging to the same contact site class as the query sequences were considered as positive, while the others were considered as negative. For each subject sequence (positive or negative), the highest scoring hit (in terms of bit score [136]) to a query sequence was recorded. Where BLASTP did not find any hit (despite practically unlimited E‐value), a bit score equal to zero was assigned. Subject sequences were ranked according to bit scores so that ROC curves could be calculated analogously to usage of grammar scores in the case of PCFGs.

Our PCFGs and Profile HMMs can be regarded as representations of a Multiple Sequence Alignment (MSA) augmented by the ability to generalize. Thus, it is interesting to compare their performance with an MSA‐based strategy, even though the latter is computationally inefficient and difficult to apply in a real world setting. Nevertheless, in our test setting such approach could be used as a reference method since all sequences in each contact site class were (gaplessly) aligned to their class centroid on the basis of 3‐d coordinates. The method designed for the test consisted on finding the *closest neighbor* in the training set, in terms of pairwise sequence distance. For all subject sequences, their distance to each query sequence was calculated using EMBOSS’ (version 6.3.1) [137] implementation of Phylip’s [138,139] PROTDIST application (PHYLIPNEW 3.69) and Hennikof/Tyler PMB matrix [140]. For each subject sequence, the closest hit (in terms of distance) to a query sequence was recorded. Rankings according to distances were used to calculate ROC curves.

Note that we intentionally use terms “query” and “subject” instead of “training” and “test” to mark that both methods do not require training but instead rely on a reference set (“query”).

Profile HMMs are the state of the art for protein sequence matching. Unlike the two previous approaches, but similarly to our method, they require a training step consisting of statistical estimations [24]. From a theoretical point of view, Profile HMMs are approximately equivalent to probabilistic regular grammars [141] and hence cannot represent medium‐ or long‐range dependencies. HMMER is the classical Profile HMM package available from http://hmmer.org[24,25]. In this section we test both the recent HMMER 3.0 and HMMER 2.3.2 since it allows *local* (option *‐s*) and *global* (option *‐g*) search. If HMMER did not find any hit (despite practically unlimited E‐value), the bit score equal to ‐1000 was assigned. ROC curves were calculated according to rankings established from bit scores.

#### Processing

In our previous tests, sequences were preprocessed so that fragments from each helix of the pair were flanked by special symbols that were exploited by our method (see a rule for non‐terminal *Turn* in Eq.22). This information is redundant for the closest neighbor method which is based on complete alignments. On the contrary, the flanking could have been potentially useful for BLASTP and HMMER, however non‐FASTA symbols had to be removed from datasets processed by these tools. Thus, the BLASTP and HMMER methods worked on concatenated sequences of helix fragments. In addition, we tested an approach where after initial processing of each helix independently, bit scores for *two parts* (fragments) forming the pair were summed. Moreover, it was possible to modify HMMER 2.3.2 source code to allow a special *divider* symbol between helix fragments; thus the amount of alignment information used by the tool was identical to that exploited by our grammars.

**Figure 3:**
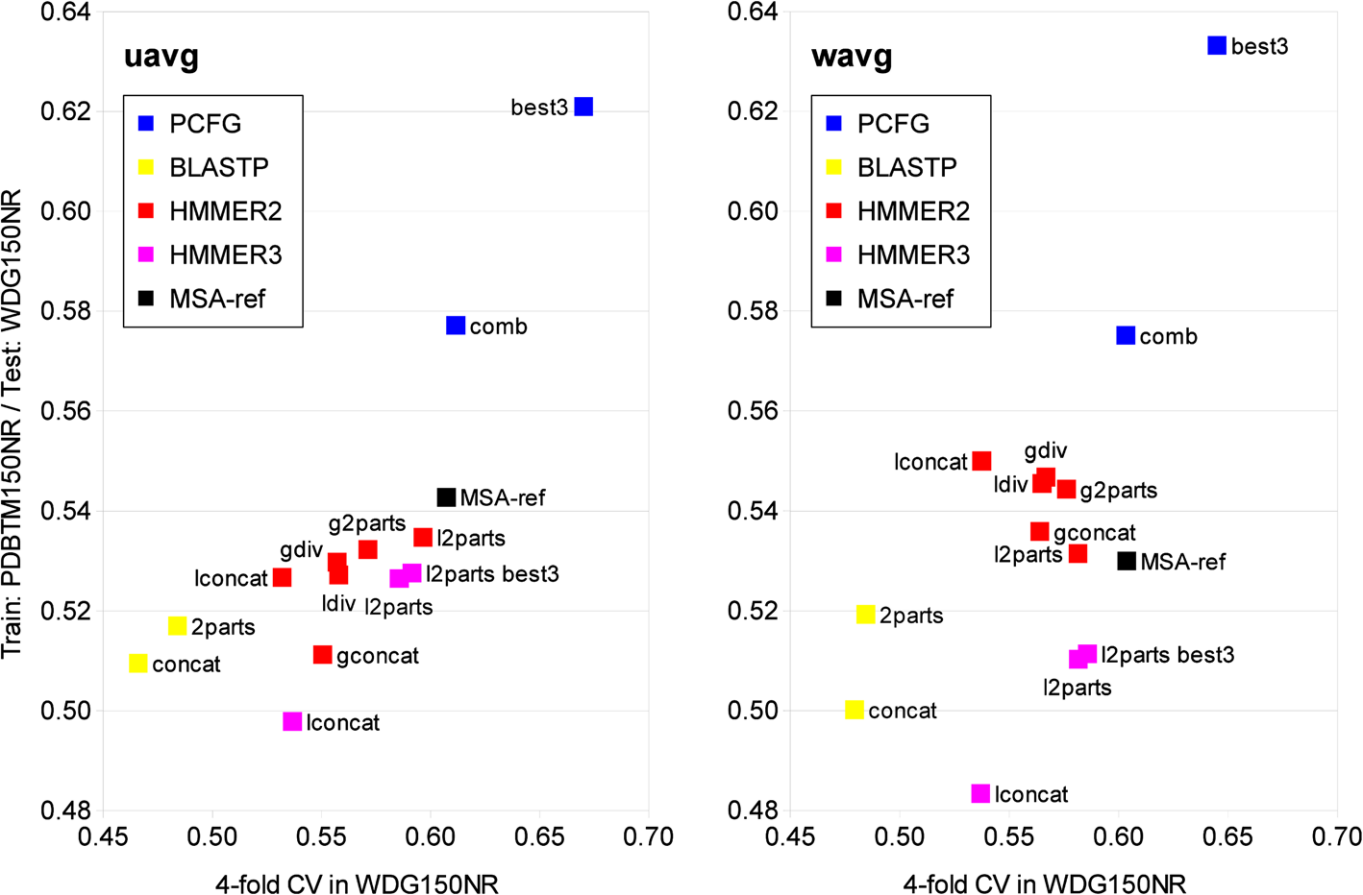
**Performance comparison with standard methods.** Average AUC ROC of classifiers in 4‐fold CV (horizontal axis) and when trained in the *PDBTM150NR* dataset and tested in *WDG150NR* dataset (vertical axis). **Left**: unweighted average, **right**: weighted average of AUC ROC. Sample method designations: **gconcat** ‐ **g**lobal HMM/**concat**ated helices scanned together; **l2parts best3** ‐ **l**ocal HMM/**2 parts** of a helix pair scanned independently; **best3** ‐ **best** classifiers out of **3**trained. In the case of the PCFGs, accessibility grammars are used for c1 and c4, and van der Waals grammars are used for c2 and c3.

The tests were carried out for 4‐fold CV in *WDG150NR* and two combinations of training and testing on*WDG150NR*and *PDBTM150NR*. HMMER3, whose training and searching steps are stochastic (*hmmbuild*and *hmmsearch*option *–seed 0*), was run three times for each set of data and options. Although HMMER2 calibration step is in principle stochastic (by default *seed=time()*), we did not see any variability of results between runs. Therefore, the tool was only run once per configuration, as BLASTP and the closest neighbor approaches which did not require any training. Results were evaluated in terms of *AUCROC* (Table 6, Table 7 and Figure 3). Average performance of best PCFGs and best HMMER3 machines, selected after testing out 3 trained classifiers (*best of 3*), is included to compare the potential of the methods. Note that performance of the combined single grammars, obtained in 4‐fold CV, was recalculated as in the case of comparison with multiple grammar classifiers and classical methods.

**Table 6:** **Comparison of classifiers performance in 4‐fold CV in the** ***WDG150NR* dataset in terms of** ***AUC ROC***

**Method/trained for**	**c1**	**c2**	**c3**	**c4**	**wavg**	**uavg**
Our PCFG comb	0.56	0.67	**0.64**	0.57	**0.60**	**0.61**
*Our PCFG best of 3*	*0.59*	*0.68*	*0.71*	*0.70*	*0.65*	*0.67*
BLASTP concat	0.51	0.45	0.47	0.44	0.48	0.47
BLASTP 2‐parts	0.49	0.47	0.51	0.47	0.48	0.48
HMMER2 global concat	0.55	0.64	0.56	0.46	0.56	0.55
HMMER2 global 2‐parts	0.56	0.61	0.62	0.49	0.58	0.57
HMMER2 global divider	0.55	0.62	0.60	0.45	0.57	0.56
HMMER2 local concat	0.54	0.58	0.45	0.56	0.54	0.53
HMMER2 local 2‐parts	0.53	0.64	**0.64**	0.57	0.58	0.60
HMMER2 local divider	0.55	0.61	0.62	0.46	0.57	0.56
HMMER3 local concat	0.52	0.57	0.53	0.52	0.54	0.54
HMMER3 local 2‐parts	**0.57**	0.61	0.57	**0.60**	0.58	0.59
*HMMER3 local 2‐parts best of 3*	*0.57*	*0.61*	*0.57*	*0.61*	*0.59*	*0.59*
MSA closest neighbor	0.56	**0.69**	0.62	0.55	**0.60**	**0.61**

*wavg* and *uavg* are *weighted* and *unweighted* averages, respectively. In the case of our PCFGs, accessibility grammars are used for c1 and c4, and van der Waals grammars are used for c2 and c3. Column best results are shown in bold (PCFG’s and HMMER3’s *best of 3* results are not considered).

**Table 7:** **Comparison of classifiers performance trained in the *PDBTM150NR *** **dataset and tested in *WDG150NR *** **dataset, in terms of *AUC ROC***

**Method/trained for**	**c1**	**c2**	**c3**	**c4**	**wavg**	**uavg**
Our PCFG comb	**0.56**	0.60	**0.59**	0.56	**0.58**	**0.58**
*Our PCFG best of 3*	*0.58*	*0.62*	*0.65*	*0.63*	*0.63*	*0.62*
BLASTP concat	0.48	0.49	0.58	0.49	0.50	0.51
BLASTP 2‐parts	0.52	0.52	0.50	0.52	0.52	0.52
HMMER2 global concat	0.55	0.61	0.44	0.45	0.54	0.51
HMMER2 global 2‐parts	0.54	0.60	0.52	0.47	0.54	0.53
HMMER2 global divider	0.55	0.61	0.50	0.46	0.55	0.53
HMMER2 local concat	**0.56**	0.57	0.58	0.39	0.55	0.53
HMMER2 local 2‐parts	0.50	0.60	0.55	0.49	0.53	0.53
HMMER2 local divider	0.55	0.61	0.50	0.45	0.55	0.53
HMMER3 local concat	0.45	0.50	0.54	0.50	0.48	0.50
HMMER3 local 2‐parts	0.48	0.53	0.54	0.56	0.51	0.53
*HMMER3 local 2‐parts best of 3*	0.48	0.53	0.54	0.56	0.51	0.53
MSA closest neighbor	0.49	**0.64**	0.42	**0.62**	0.53	0.54

*wavg* and *uavg* are *weighted* and *unweighted* averages, respectively. In the case of our PCFGs, accessibility grammars are used for c1 and c4, and van der Waals grammars are used for c2 and c3. Column best results are shown in bold (our PCFG *best of 3* is not considered).

#### Results

In the 4‐fold CV (Table 6), the best average performance achieved by combined PCFGs (0.60‐0.61) matched the reference results of the closest neighbor approach. They were closely followed by HMMER2 *local 2‐parts*(0.58‐0.60) and HMMER3 *local 2‐parts*(0.58‐0.59). Apparently, local HMMs performed better when sequences were split in 2 parts (ca. 10% improvement). In the case of *WDG150NR*training and *PDBTM150NR*testing all methods performed poorly (not shown). Best average *AUCROC*s were 0.53‐0.55 for HMMER2 *divider*and 0.52‐0.55 for combined PCFGs. *AUCROC*s over 0.60 were achieved only by HMMER3 (0.60‐0.62) and HMMER2 *global concat*(0.61) for class 4 and by combined PCFG for class 3 (0.60). Eventually, *PDBTM150NR*trained classifiers were tested in *WDG150NR* (Table 7). In this setup, the combined PCFG was the best method with average *AUCROC* of 0.58. It was followed by HMMER2 (up to 0.53‐0.55) and the closest neighbor reference method (0.53‐0.54). Surprisingly, HMMER3 *local 2‐parts*performed very poorly (0.51‐0.53).

In virtually all cases BLASTP achieved *AUCROC* close to 0.50, because it was able to assign a class only to a fraction of subject sequences. For example, BLASTP *concat* found matches to 48‐82% of helix pairs in 4‐fold CV and to 24‐75% of helix pairs in the *PDBTM150NR*training and *WDG150NR* testing. This demonstrates that the helix‐helix contact site classification problem is out of scope of a simple sequence similarity search and requires a more powerful solution. Figure 3 shows that combined PCFGs outperformed Profile HMMs from HMMER packages, especially when trained on the *PDBTM150NR* dataset. In the most direct comparison, the combined PCFGs were better than HMMER2s *divider*by 5 to 10% in both dataset configurations. In the*PDBTM150NR*training and *WDG150NR* testing setups, the combined grammar classifiers outscored HMMER3 *local 2‐parts*by ca. 10%. Moreover, in the same setups, PCFG *best of 3*achieved about 20% larger *AUCROC* than HMMER3 *local 2‐parts best of 3.* Finally, performance of PCFGs and Profile HMMs are compared to results of the MSA‐based strategy. In the case of 4‐fold CV, the combined grammars and the HMMs matched the closest neighbor reference approach despite lacking complete alignment information. In the other setup, PCFGs had 6‐8% larger *AUCROC* than the MSA‐based reference strategy due to significantly better recognition of class 3 (by 41%). This illustrates the ability of grammar induction to generalize over the training sample.

### Analysis and discussion

We have demonstrated the capability of grammar classifiers to learn helix‐helix contact site classes from sequences in *theWDG150NR* dataset. The results were consistent between LOO and 4‐fold CV. While the average performance in terms of *AUCROC* was moderate (approximately 0.60), this improved to 0.65 when the helix pair classification problem was treated explicitely as a 4‐way classification (multiple grammar classifiers, Table 4). Testing in the independent *PDBTM150NR* dataset has shown 20% worse performance. This could indicate that sequence based features, distinctive to the four helix‐helix contact site classes, may not be as well defined in the *PDBTM150NR* as in the original *WDG150NR* dataset (see also Additional file 1: Figure S1). We demonstrated that the helix‐helix contact site classification problem cannot be solved by a simple BLAST search and requires a more powerful solution. The combined grammar classifiers outperformed state‐of‐the‐art Profile HMMs from the HMMER2 and HMMER3 packages in the 4‐fold CV in *WDG150NR* dataset. Moreover, our PCFG method was the only which learnt from *PDBTM150NR* to classify the *WDG150NR* sample for all four contact site classes. Comparison with reference results of the MSA‐based closest neighbor in the training set approach demonstrated the grammar induction ability of generalization over the training sample.

In the next section, we will investigate the foundations of grammar classifiers performance and their relations to helix pair features. Moreover, correspondence between grammar structures and spatial configuration of helix pairs is assessed.

#### Added value of grammar classifiers

Better performance of the grammar classifiers in relation to Profile HMMs can be related to (1) higher expressive power allowing (2) problem‐specific grammar structure, but also (3) usage of amino acid properties and (4) selection of the most informative of them for a given class (which requires a validation step). Impact of the first two features was expected to be seen especially in the anti‐parallel contact site classes 1 and 2, where dependencies between residues can be directly modelled by a PCFG. However, the combined grammars are better than HMMs only for class 2 in the 4‐fold CV. Moreover, even higher *AUCROC* of the reference closest neighbor method for that case suggests that good performance can also be grounded in sequence similarity. On the other hand, usage of amino acid properties by the PCFGs considerably reduces sequence information in comparison to that utilised by the other methods. Thus, it is possible that higher order dependencies represented by the grammars compensates this. Best single PCFGs significantly outperformed other methods even in class 3 and class 4 in the 4‐fold CV. However, these results cannot be directly linked with modelling of nested dependencies as those contact site classes are parallel. Further in this section, we investigate the hypothesis that performance of the grammar classifiers is mainly grounded in the average value of the amino acid property level.

Let the average property level classifier assign a contact site class on the basis of the average amino acid property level in the helix‐helix sequence (Table 8). Its training only consists on establishing a preference towards higher or lower property level based on positive and negative training sets. For the purpose of comparison with the average property level classifier, performance of the combined single grammars, obtained in 4‐fold CV, was recalculated as in the case of comparison with multiple grammar classifiers and standard methods.

**Table 8:** Added value of grammar classifiers

**Trained for**	**Accessibility**		**vdW volume**	
	**Avg. prop.**	**Grammar**	**Avg. prop.**	**Grammar**
c1	0.44	0.56(0.59)	0.58	0.41 (0.45)
c2	0.43	0.45 (0.50)	0.61	0.67(0.68)
c3	0.61	0.49 (0.55)	0.64	0.64(0.71)
c4	0.62	0.57 (0.70)	0.69	0.44 (0.55)

Helix‐helix contact fragments classification in the *WDG150NR* dataset on the basis of amino acid property average and using the combined grammars. Average AUC ROC of the best performing classifiers, out of 3 trained grammars for each class‐property case, is shown in parentheses.

We found that *AUCROC*s of the best single grammar classifiers were better than *AUCROC*s of the average property level classifiers by 0.07‐0.15 (11‐34%) for accessibility grammars of classes 1 and 4, and for van der Waals volume grammar of classes 2 and 3. These results suggest rejecting the hypothesis that performance of the grammar classifiers is mainly grounded in the average value of the amino acid property level and support the idea that grammar capability to represent intra‐ and/or inter‐ helical dependencies contributes to successful classification. Moreover, we found that combined grammar classifiers performed better than the average property level classifiers for the accessibility grammar of class 1 and van der Waals volume grammar of class 2.

No good grammar classifier based on the van der Waals volume property was obtained for class 1 and 4, despite relatively good performance of the average property level classifiers. Similar was the case of the accessibility property and class 3. It is likely that in these cases grammars learnt features of *other* classes rather than the class for which they were trained. For example, the combined grammar classifier based on the van der Waals volume property trained for class 1 achieved *AUCROC* of 0.41. Interestingly, if it was applied to distinguishing the class 3 pairs from pairs in classes 1, 2 and 4, its *AUCROC* would be relatively high 0.66 (not shown). We hypothesized that, perhaps, some of the helix‐helix contact site classes shared the same features, related to amino acid accessibility or van der Waals volume. However, while one feature was dominant in one class, it was only weakly present in the other. Nevertheless, that weak presence of the feature could be learnt by a grammar in our framework. As a result, such a grammar would rather fit the class where the feature was dominant rather than the class for which it was trained.

Finally, we studied which of the PCFG capabilities contribute to its overall performance. For this purpose, grammars incapable of directly representing inter‐helical contacts were designed and their rule probabilities were inferred.

First, we performed several modifications of our original helix‐helix grammar so that the 3rd RHS symbol (*Single* or *Double*) was removed from the rules rewriting *Inside* and *Outside* non‐terminals to break inter‐helical dependencies. Moreover, *Turn*→ *Whatever* ] { *WhateverInside*/*Outside* rules were added to account for two helices and *Inside*/*Outside*→*ϵ* to finish the derivation. In this way, the rule set continued to account for helix periodicity and boundaries of helix fragments. In comparison to our original PCFGs, combined classifiers based on the modified helix‐helix grammars matched the original PCFGs in *WDG150NR* (*AUCROC* 0.56‐0.61). Moreover, the modified grammars were even better than the combined original helix‐helix PCFGs in the *PDBTM150NR*training /*WDG150NR*testing (0.57‐0.61); however they obtained very poor scores for class 1 (below 0.53).

Second, we produced grammars whose (otherwise unconstrained) rules allowed only adding one property non‐terminal *A*∈{*Low*, *Medium*, *High*, *Any*, *Turn*} at each derivation step. Thus, the rule set did not include any prior knowledge about protein fragments. Combined classifiers based on the unconstrained grammars achieved slightly worse average *AUCROC*s in the 4‐fold CV in *WDG150NR* (0.56‐0.59) and comparable scores in the *PDBTM150NR*training /*WDG150NR*testing scheme (0.57‐0.58). The best single unconstrained grammars performed worse than the best single original helix‐helix PCFGs.

These results indicated that direct representation of inter‐helical dependencies was not required for achieving maximal performances reported in this study. This finding is consistent with the fact that PCFGs trained for parallel and anti‐parallel contact site classes worked similarly well. Interestingly, the most successful types of grammars contained rules dealing with helix periodicity, which could be represented practically (i.e. using reasonable number of rules) only in the context‐free framework. Consequently, this capability seems to boost the PCFG performance in our framework.

#### Correlation between helix pair features and grammar classifier efficiency

Correlation between performance of single grammar classifiers and helix pair structure and sequence was analysed using the single grammars generated in the LOOCV scheme. Three features were considered: 

• Spatial distance in terms of *RMSD* in 3D, calculated relatively to the class centroid,

• Average normalized distance *dist* to other class members in terms of sequence similarity. All distances, calculated by ClustalW 1.83 [142], ranged from 0.87 to 0.95.

• *multiplicity* of helix pair contacts, i.e. a number of other helices in contact with the helix pair of interest.

Intuitively, the correlation between grammar classifier performance and all three features is expected to be near zero or negative, e.g., the more helix‐pair differs from its class average in terms of sequence or spatial similarity, the lower *AUCROC* is expected. Moreover, if a helix pair is involved in other helix‐helix contacts, then its sequence and spatial conformation had to adjust to these other contacts. Thus, the helix pair structure and sequence would diverge from the pair‐wise mean for a given class.

In this study, the correlation between *AUCROC* of single grammar classifiers and features of helix pair structure and sequence were represented by Pearson’s R coefficient (Table 9).

**Table 9:** Correlation between helix pair features and grammar classifier efficiency

**Trained for**	**Using property**	**RMSD**	**Dist**	**Multiplicity**
c1	accessibility	‐0.08	‐0.14	‐0.14
c2	vdW volume	+0.16	**‐0.45**	‐0.25
c3	vdW volume	‐0.44*	**‐0.62**	‐0.24
c4	accessibility	‐0.30	‐0.47	‐0.12

Pearson’s R correlation coefficients values between *AUC ROC* and some helix pair features, i.e. spatial distance (*RMSD*), sequence alignment distance (*dist*) and helix contact *multiplicity*. Only Classifiers built using the most informative amino acid properties for a given class are shown. P‐values below 0.05 are shown in bold. *not confirmed by Spearman rank correlation.

In agreement with expectations, the performance of grammar classifiers was negatively correlated with the sequence alignment distance. However, the observed linear correlations were statistically significant at p‐value below 0.05 only in the case the van der Waals grammars of classes 2 and 3 (Table 9). The linear correlation of *AUCROC* and helix contact *multiplicity* was always negative, yet not statistically significant. While relatively high Pearson’s R was observed between *AUCROC* of the van der Waals volume classifiers of class 3 and the spatial distances from the template (p‐value below 0.04), the dependency was not confirmed by the Spearman rank correlation. The weak positive correlation of the same features for van der Waals volume grammar of class 2 seems to be due the unexpected negative linear correlation between spatial and sequence similarities in class 2 (Pearson’s R equal to ‐0.42, not shown).

#### Analysis of parse trees of single grammar classifiers

The best performing grammar descriptors of class 1 and 2 were analysed in terms of their average *a priori* parse trees. The best single accessibility‐based grammar of class 1, pruned of low probability (Pr <0.05) rules and normalized, is shown in Eq. 26:

where, for the sake of brevity, the property of non‐terminals, i.e. *Low*, *Medium*, *High* and *Any* (Eq. 21) are denoted as *l*, *m*, *h* and *x*, respectively; *Double* non‐terminals (Eq. 20) are denoted as *A*,*B*,*C*; *Inter*- and *Outerface* non‐terminals (Eq. 22) are denoted as *T*,*U*,*V*,*W* and *O*,*P*,*Q*,*R*; *Whatever* non‐terminal is denoted as *X*, and *S* is the start symbol of the grammar (see Eq. 22).

**Figure 4:**
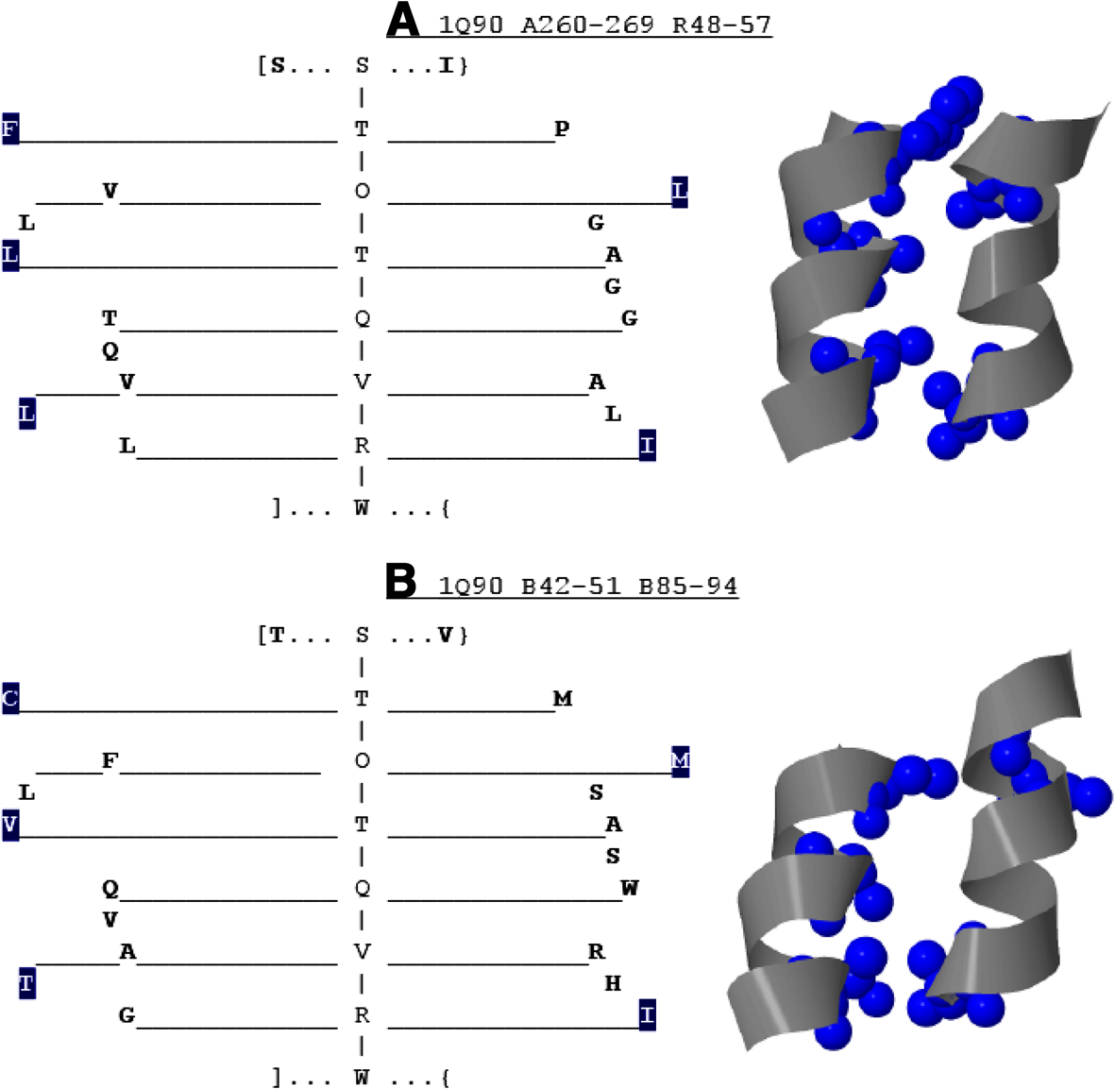
**An average parse tree of accessibility‐based grammar of class 1 (Eq.**26**).** Lengths of branches express expected level of accessibility. Terminal symbols on the tree branches represent residues of two helix pairs (panels **A,B**) from chain A of cytochrome b6f from C.reinhardtii (PDB code: 1Q90). The residues expected to be the most accessible, according to the grammar, are shown in blue in the parse tree and as blue balls in the structural model of the helix‐helix contact site. The acceptance rate of PDBTM helix pairs assignment to a given WDG contact site class in function of the RMSD cutoff.

For this grammar, its most common parse tree stem in the training set (present in 24% of maximal parse trees) was utilized in the Viterbi parses of 2 test cases (Figure 4). The most prominent feature of the parse tree is *i*+3/*i*+4 periodicity of highly accessible residues in the left helix. In the right helix, two highly accessible amino acids are separated by 7 less accessible residues. Interestingly, highly accessible residues are pointed towards the other helix (except methionine in Figure 4B) in the manner resembling the knobs‐into‐holes configuration [84,85].

**Figure 5:**
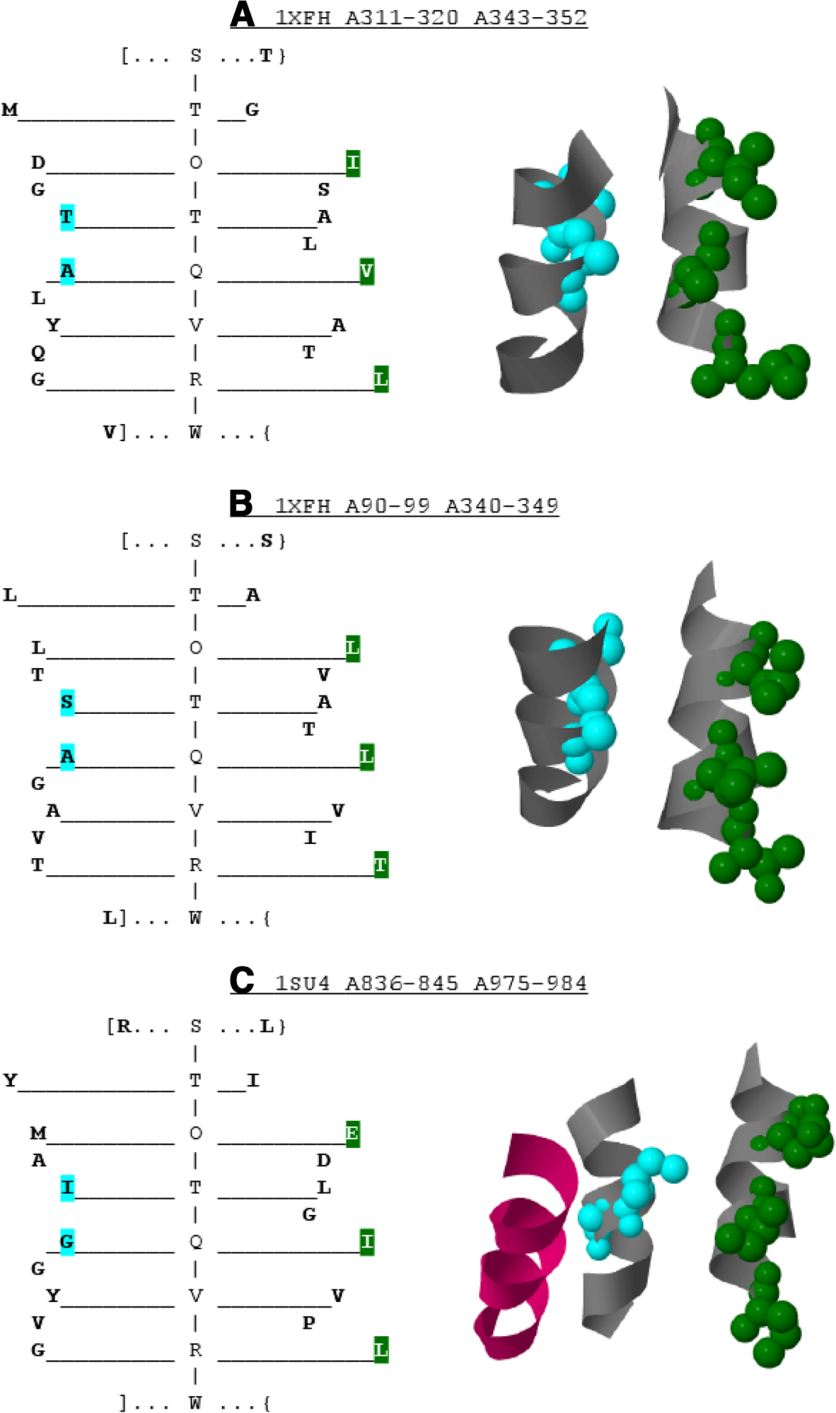
**An average parse tree of van der Waals‐based grammar of class 2 (Eq.**27**).** Lengths of branches express expected level of van der Waals volume. Terminal symbols on the tree branches represent residues of two helix pairs from: **(A,B)** glutamate transporter from P.horikoshii (PDB code: 1XFH) and **(C)** calcium ATPase from O.cuniculus (PDB code: 1SU4). The residues expected to be the smallest in the left‐hand‐side helix, according to the grammar, are shown in cyan. The residues expected to be the largest in the right‐hand‐side helix, according to the grammar, are shown in green. Note, that the helix contact in (C) is disturbed by the presence of another helix (drawn in magenta).

The best single van der Waals‐based grammar of class 2, pruned of low probability (Pr <0.05) rules and normalized, is shown in Eq. 27: 

For this grammar, its maximal parse tree for 3 test cases (Figure 5) based on the second most common stem in the training set (33% of Viterbi parses) is shown. Positions of the two smallest residues in the left helix indicate surface of the flat contact. On the other hand, large residues in the right helix (peridicity *i*+3/*i*+4) are on the non‐contacting side of the helix. Note, that the helix contact in Figure 5C is disturbed by the presence of another helix (drawn in magenta). This fact was reflected by lower probability of the Viterbi parse for that case.

This analysis shows that the grammar parse trees can convey biologically meaningful information regarding structural features of helix‐helix contact sites.

#### Explicit multi‐class classification

Walters and DeGrado [91] identified 14 classes of transmembrane helix‐helix pairs. In our study, we only considered the 4 most populous classes, because the other classes did not include sufficient number of members for automated training of classifiers. Therefore, our principle approach was to train classifiers independently for each class, which does not assume knowledge of the entire spectrum of possible helix‐helix configurations. However, we also studied the approach where the the helix pair classification problem was treated explicitely as a multi‐class classification. Multiple grammar classifiers were generated to solve a 4‐way problem of assigning a helix pair to one of the four most populous classes. This resulted in the average *AUCROC* by almost 10% higher than for combined grammars (Table 4) as a benefit of combining positive and negative information through averaging over scores of grammars trained for each class (Eq. 23). For the same reason, performance of the multiple grammar was approximately even for all the helix‐helix pair classes. The multiple grammar classifiers would be therefore preferable to single or combined grammars if a larger dataset of helix pairs, encompassing the entire spectrum of their configurations, was available for training (see Limitations below).

#### Limitations

##### Context‐free grammars

PCFG, used in this research, are able to represent explicitly nested dependecies between residues formed by anti‐parallel helices (e.g. class 1 and 2). However, associations of residues in a pair of parallel helices (classes 3 and 4) form crossing dependencies, which can be explicitly described only by context‐sensitive rules. Parsing of the context‐sensitive languages generally has exponential time complexity, which can be reduced to higher order polynomial complexity for some subclasses, e.g. *O*(*n*^6^) in the case of mildly context‐sensitive languages [143,144]. Instead of time consuming inference of probabilities of mildly context‐sensitive rules, we opted for learning probabilities of context‐free rules for parallel helix pairs, which linked residues at the opposing sites of helices. Therefore, grammar descriptors generated for classes 3 and 4 cannot be directly interpreted as in the case of anti‐parallel pairs. Alternative strategy could rely on reversing of sequence direction in one helix of the parallel pair. Then, for an unknown pair of helices, both cases (normal and reversed direction of one of the helices) could be evaluated.

##### Learning scheme

Noticeable better performance of the best single grammars, in comparison to combined (averaged) grammars (Tables 9 and 5), suggests that our framework could benefit from further development of the evolutionary inference scheme in order to assure obtaining solutions consistently close to the global optimum. As an alternative, limitations of the current grammar induction process could be complemented by selecting the best grammar classifier from several trained candidates by means of validation, especially if more comprehensive datasets are available.

##### Dataset

The Walters and DeGrado [91] classification of helical pairs in transmembrane proteins was the first attempt to systematic identification of helix‐packing geometries with their encoding sequence motifs. Moreover, despite recent interest in helix packing in transmembrane proteins, their approach has not been superceded by up‐to‐date works [90,145]‐ [147]. Therefore, it was natural do adopt the WDG classification as the basis for our research. An apparent limitation of this dataset is its relatively small size. This did not allow us to induce grammar classifiers for less populous WDG classes of helix‐helix arrangements which contain 1/3 of the sample. Moreover, interpretation of results for small classes 3 and 4 is less dependable. Therefore, we attempted to enlarge the available dataset using PDBTM database [47]. However, we noticed that coherence of PDBTM‐based dataset with WDG classification in terms of spatial similarity did not implicate coherence in terms of sequence features (see Additional file 1: Figure S1 and Additional file 3: Figure S3). Therefore, generation of a new larger set of helix‐helix pairs could require to redefine helix‐helix pair classification.

## Conclusions

We presented an original probabilistic grammatical model of a protein language. The model covers the lexical (primary structure) and syntactical (secondary and tertiary structure) levels of protein linguistics. The core of the model consists of a probabilistic context‐free grammar, whose rule probabilities are automatically inferred by a genetic algorithm from positive training samples only. In this process, an initially large generic set of rules is reduced to include only those rules which are relevant. We demonstrated the capability of the context‐free framework for analysis of protein sequences. Sequence based grammar descriptors, which represented four classes of transmembrane helix‐helix contact configurations, defined by Walters and DeGrado [91], were induced and tested using cross‐validation on a modified non‐reduntant version of their original dataset [91]. The predictive power was assessed in class‐of‐interest *versus* other classes classification. The quality of the grammar classifiers was primarily evaluated in terms of Area Under ROC curves. The highest performance of the combined grammars (averaging over three single grammars) reached *AUCROC* of 0.68 (average over the four classes 0.60). Moreover, best single grammars achieved *AUCROC* of 0.71 (average over the four classes 0.67). In addition, when the helix pair classification problem was treated explicitely as a 4‐way problem of assigning a helix pair to one of the four most populous classes, our multiple grammar classifiers achieved an average *AUCROC* of 0.65.

The grammar classifiers were then tested on a independent dataset obtained from the PDBTM database [47]. The performance was 20% lower (best *AUCROC* of 0.60). We found that the sequence‐based features distinctive to the four helix‐helix contact classes in the Walters and DeGrado dataset are likely to be less well defined in the PDBTM‐derived dataset. However, when PDBTM‐derived dataset was used for training and WDG‐derived dataset served as the test sample, grammar classifiers achieved an average *AUCROC* of 0.58. This result is significant since all standard approaches, including Profile HMMs, obtained in this setting *AUCROC* below 0.55. The advantage of our probabilistic context‐free framework for analysis of protein sequences over the current state of the art seems to be grounded in representing helix periodicity. While this feature does not require modeling long range dependencies, its implementation is facilitated by the context‐free framework.

A significant feature of our approach is that the grammar rules and parse trees are human‐readable [37]. In this paper we introduced the notion of the average *a priori* parse tree as a tool for convenient elucidation of biological information from a probablistic grammar representation of a protein fragment. Analysis of the sample trees (Figures 4 and 5) suggests that PCFGs whose rule probabilities were induced automatically, could represent biologically meaningful features of protein structures only based on amino‐acid sequence.

A possible future application of our method is residue‐residue contact prediction based on residue‐residue dependencies derived from the parse tree of a helix sequence. However, as the relation between grammar dependencies and real residue‐residue contacts is not straightforward, a practical approach could consist on 3 steps: (1) assigning a helix pair of unknown structure to a contact site class; (2) threading the pair on the class centroid used as a template; (3) assigning residue‐residue contacts based on the predicted structure. Our approach can also be potentially used to detect plausible helix pairings of a certain type in a given set of helices.

## Competing interests

The authors declare that they have no competing interests.

## Authors’ contributions

WD and JCN ‐ concept. WD designed methodology of the classification, developed software implementing the methodology, prepared training data and performed computational experiments. WD, JCN and MK performed data analysis. WD described results. WD, JCN and MK prepared the final manuscript. All authors have read and approved the final manuscript.

## Supplementary Material

Additional file 1 **Figure S1.** Average values of amino acid properties. Average values of amino acid properties in the four classes in *WDG150NR* (white bars) and *PDBTM150NR* (gray bars). Notation: *acc* ‐ accessibility AAindex:BIOV880101 [126];*vol* ‐ van der Waals volume AAindex:FAUJ880103 [125]). Click here for file

Additional file 2 **Figure S2.** Class centroids. Class centroids from WDG datasets cut to the length of 10‐10 residues using a criterion of the most concise geometrical representation. Click here for file

Additional file 3 **Figure S3.** PDBTM pairs assignment to WDG classes. Acceptance rate of PDBTM helix pairs assignment to a given WDG contact site class in function of the RMSD cutoff. Click here for file

Additional file 4 **Figure S4.** ROC curves in the LOOCV. The ROC curves were produced using the vertical averaging method [130] for band length 0.1. Normal approximation intervals are shown. Notation: *acc* ‐ accessibility AAindex:BIOV880101 [126]; *vol* ‐ van der Waals volume AAindex:FAUJ880103 [125]). Click here for file

Additional file 5 **Table S1.** Classification performance measures in the LOOCV after vertical averaging at selected FP rate thresholds. Click here for file

Additional file 6 **Figure S5.** Specificity in *WDG150NR* dataset in the LOOCV experiment. Each bar represents *AUCROC* of classification of a single helix pair against all helix pairs in the negative sample. White bars indicate *AUCROC* greater or equal to 0.75. Notation *c1/c2+c3+c4* means that the grammar was trained for class *c1* and then tested for class *c1* against three other classes *c2‐c4*. Click here for file

Additional file 7 **Table S2.** Classification performance measures in the 4CV, using **(a)** combined and **(b)** best single grammars, after vertical averaging at selected FP rate thresholds. Click here for file
