# Supplementary figures and images for: Probabilistic grammatical model for helix‐helix contact site classification
Source: Algorithms Mol Biol. 2013 Dec 18;8:31. doi: 10.1186/1748-7188-8-31 (PMC3892132; doi:10.1186/1748-7188-8-31)

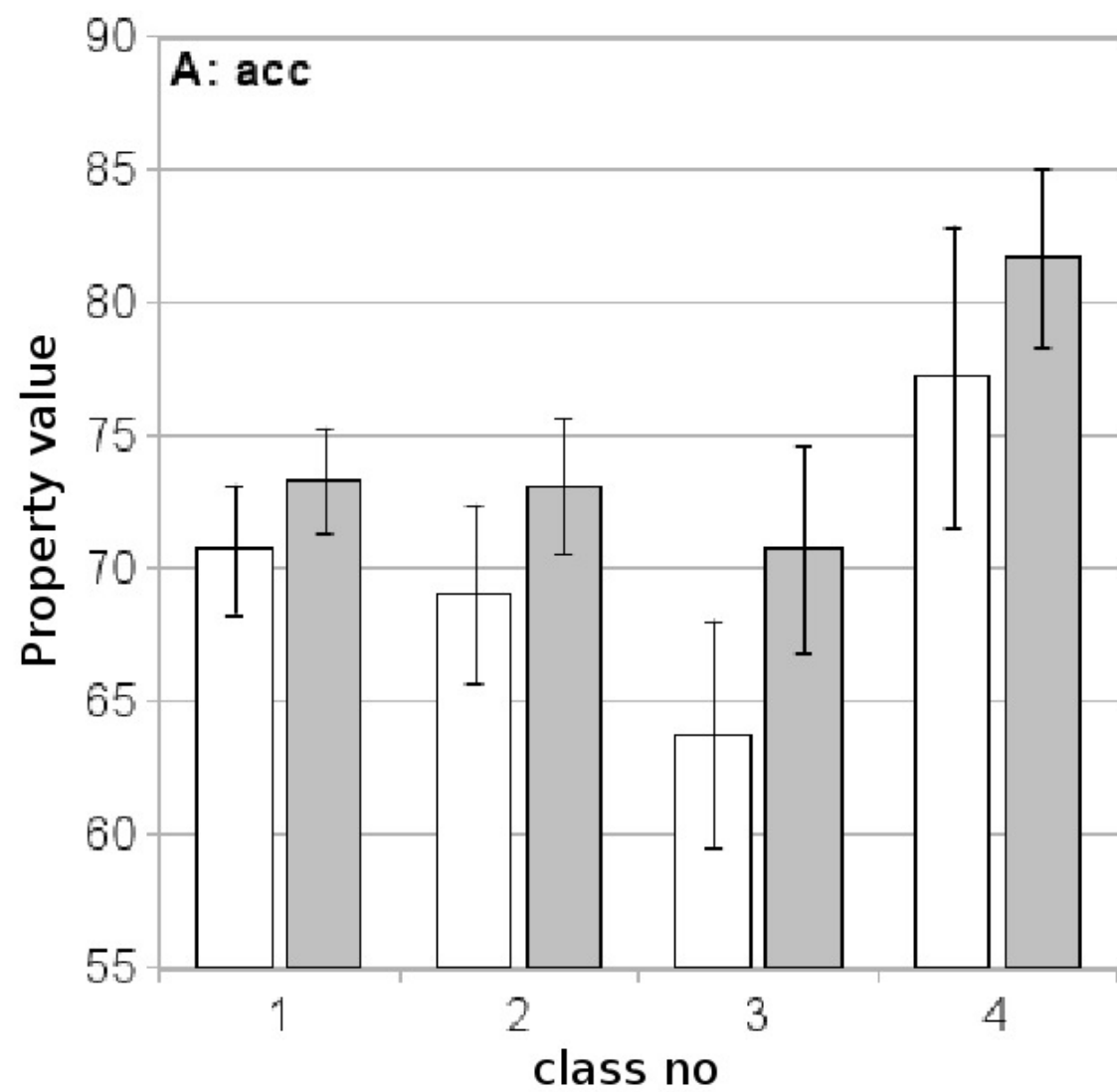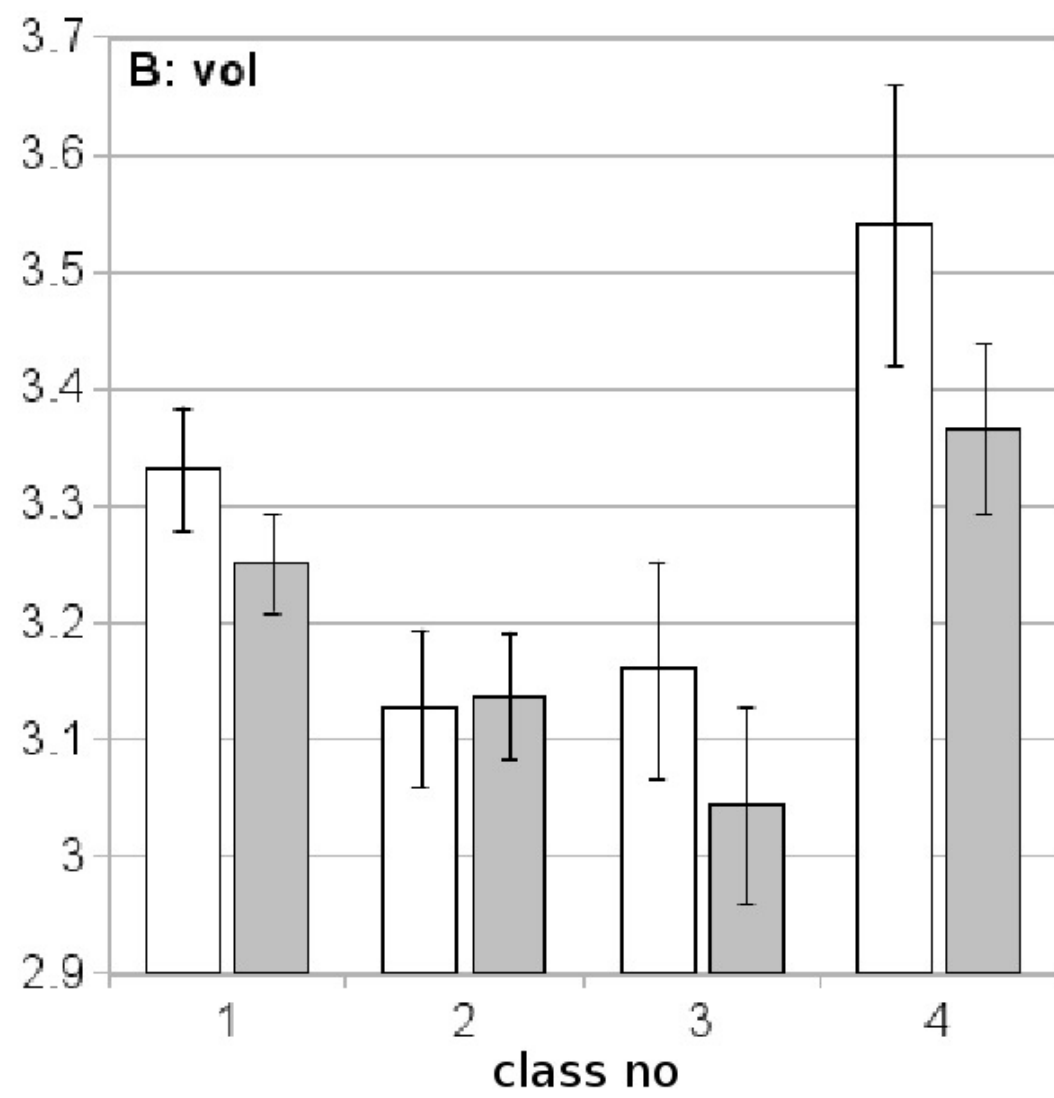

Supplement: Additional file 1 — Figure S1. Average values of amino acid properties. Average values of amino acid properties in the four classes in WDG150NR (white bars) and PDBTM150NR (gray bars). Notation: acc ‐ accessibility AAindex:BIOV880101 [126];vol ‐ van der Waals volume AAindex:FAUJ880103 [125]). [file 1748-7188-8-31-S1.pdf]

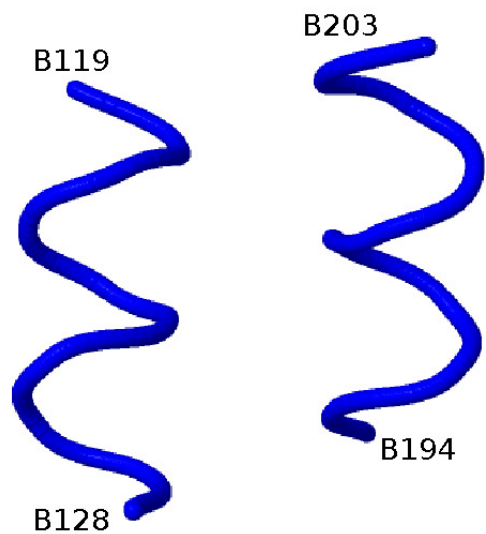

1Q90  
class 1

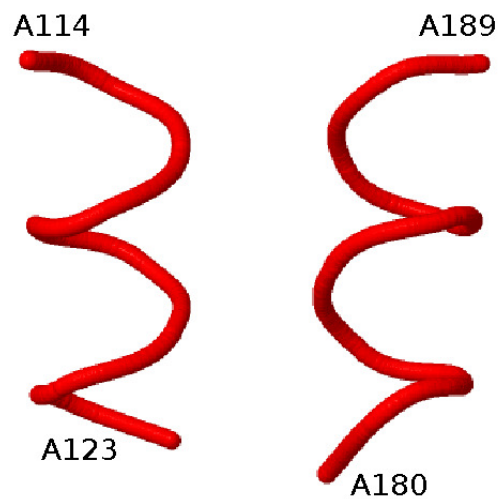

1OKC  
class 2

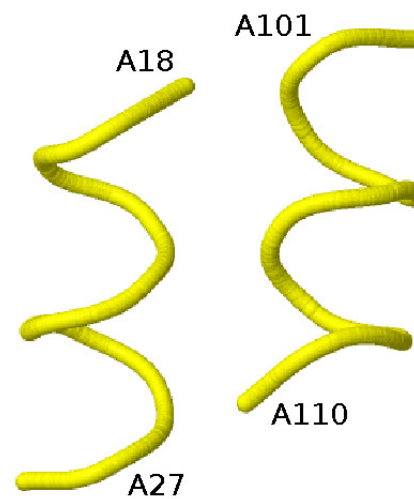

1J4N  
class 3

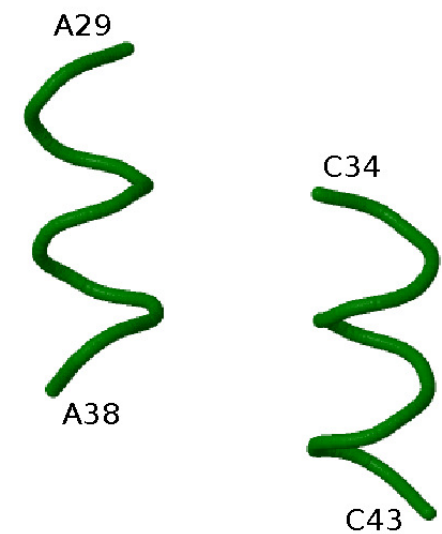

1RH5  
class 4

Supplement: Additional file 2 — Figure S2. Class centroids. Class centroids from WDG datasets cut to the length of 10‐10 residues using a criterion of the most concise geometrical representation. [file 1748-7188-8-31-S2.pdf]

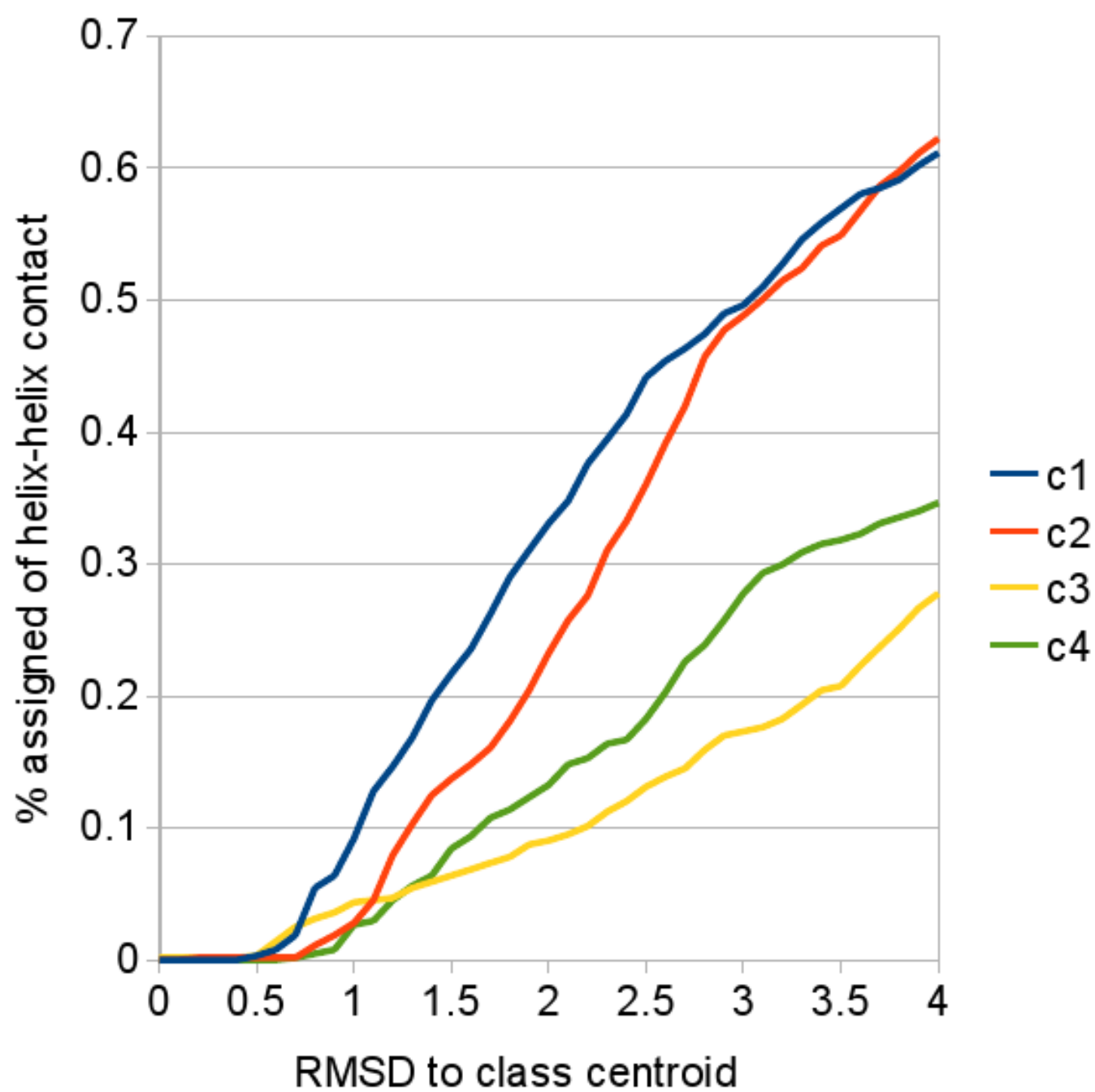

Supplement: Additional file 3 — Figure S3. PDBTM pairs assignment to WDG classes. Acceptance rate of PDBTM helix pairs assignment to a given WDG contact site class in function of the RMSD cutoff. [file 1748-7188-8-31-S3.pdf]

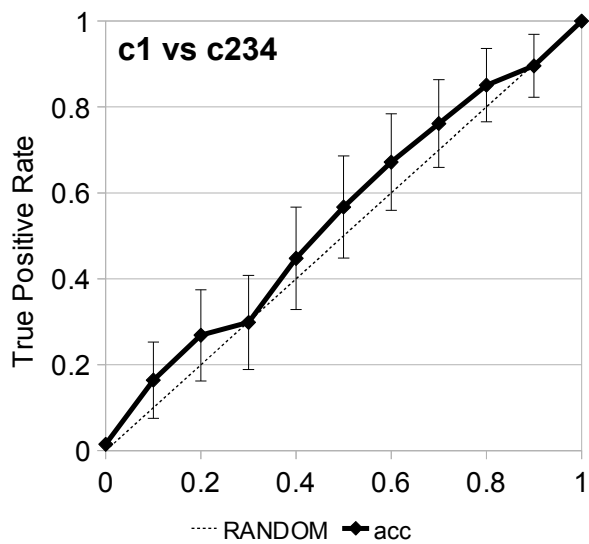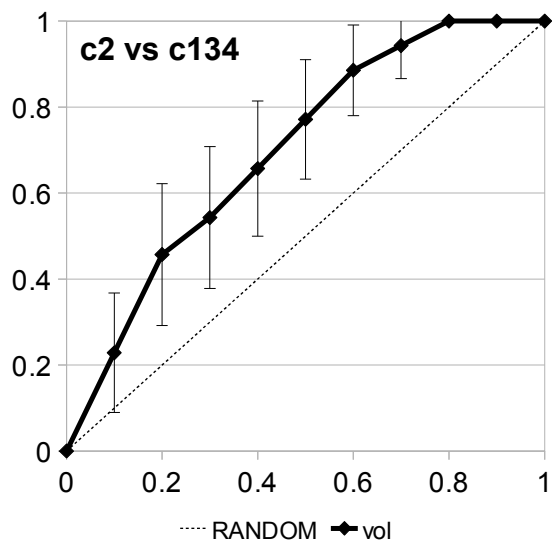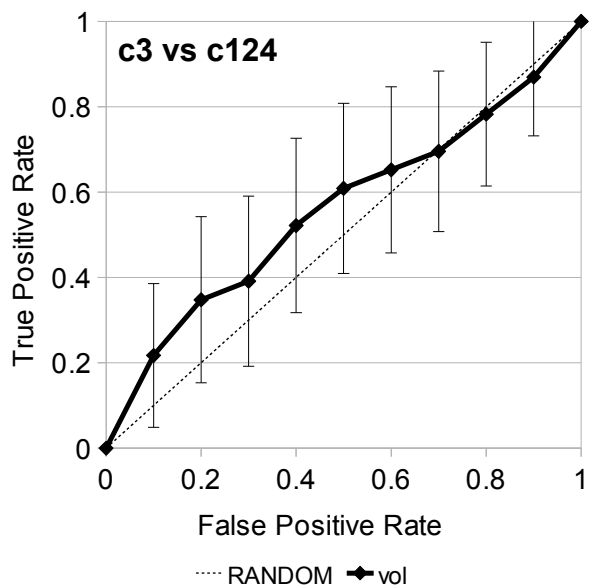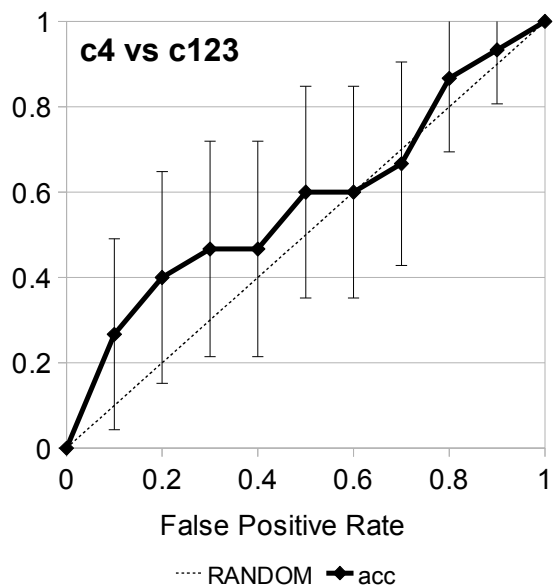

Supplement: Additional file 4 — Figure S4. ROC curves in the LOOCV. The ROC curves were produced using the vertical averaging method [130] for band length 0.1. Normal approximation intervals are shown. Notation: acc ‐ accessibility AAindex:BIOV880101 [126]; vol ‐ van der Waals volume AAindex:FAUJ880103 [125]). [file 1748-7188-8-31-S4.pdf]

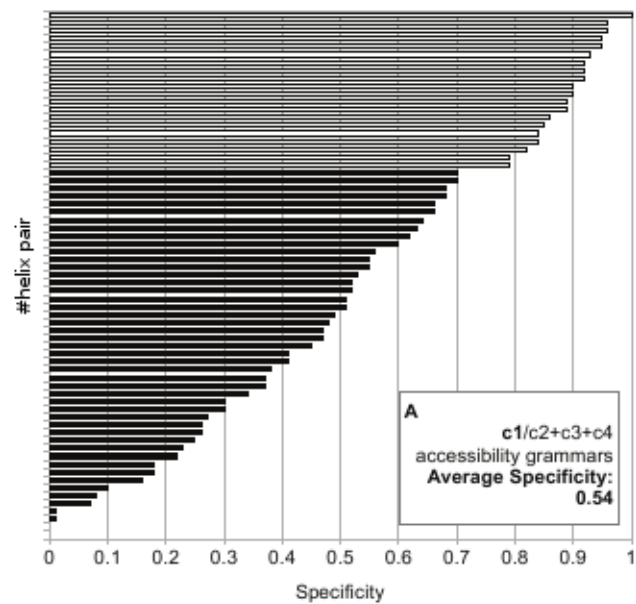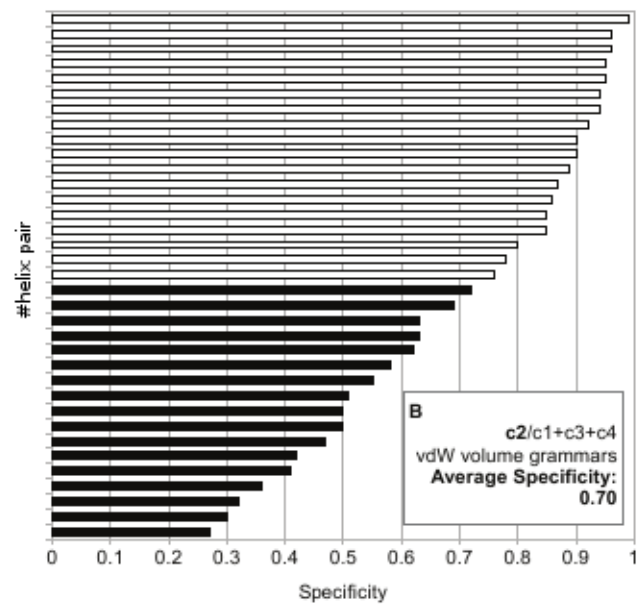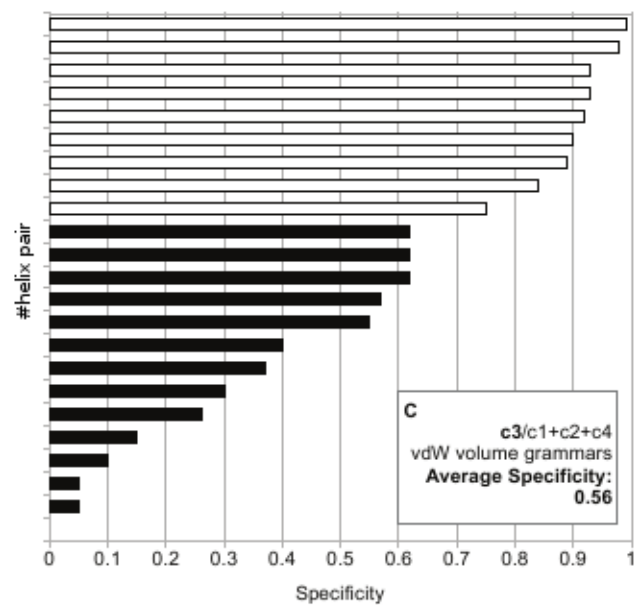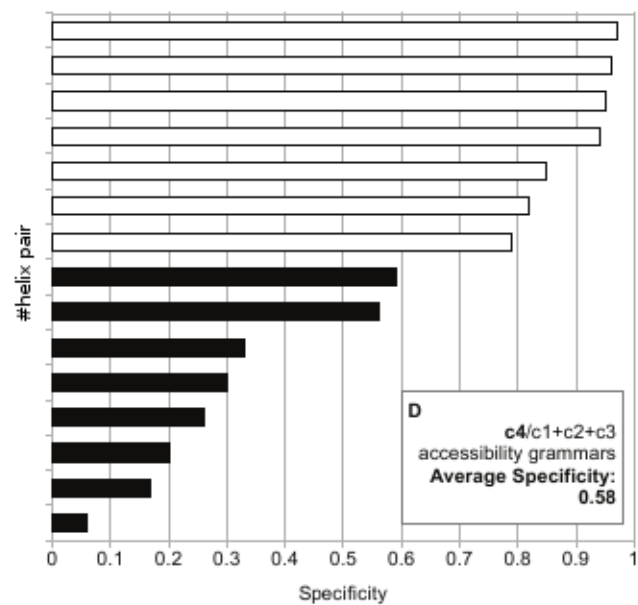

Supplement: Additional file 6 — Figure S5. Specificity in WDG150NR dataset in the LOOCV experiment. Each bar represents AUCROC of classification of a single helix pair against all helix pairs in the negative sample. White bars indicate AUCROC greater or equal to 0.75. Notation c1/c2+c3+c4 means that the grammar was trained for class c1 and then tested for class c1 against three other classes c2‐c4. [file 1748-7188-8-31-S6.pdf]
