# Supplementary material for: Probabilistic grammatical model for helix‐helix contact site classification
Source: Algorithms Mol Biol. 2013 Dec 18;8:31. doi: 10.1186/1748-7188-8-31 (PMC3892132; doi:10.1186/1748-7188-8-31)
Supplement: Additional file 5 — Table S1. Classification performance measures in the LOOCV after vertical averaging at selected FP rate thresholds. [file 1748-7188-8-31-S5.pdf]

LOOCV measures

Supplementary Table 1: Classification performance measures in the LOOCV after vertical averaging at selected FP rate thresholds

	FP rate	*Precison*	*Recall*	*F1*	*Accuracy*
c1acc	0.3	0.48	0.30	0.37	0.51
	0.5	0.51	0.57	0.54	0.53
	0.7	0.50	0.76	0.60	0.52
c2vol	0.3	0.38	0.54	0.44	0.66
	0.5	0.34	0.77	0.47	0.57
	0.7	0.31	0.94	0.47	0.46
c3vol	0.3	0.20	0.39	0.27	0.65
	0.5	0.19	0.61	0.29	0.52
	0.7	0.16	0.70	0.26	0.36
c4acc	0.3	0.16	0.47	0.24	0.68
	0.5	0.13	0.60	0.21	0.51
	0.7	0.10	0.67	0.18	0.34
