# Supplementary material for: Probabilistic grammatical model for helix‐helix contact site classification
Source: Algorithms Mol Biol. 2013 Dec 18;8:31. doi: 10.1186/1748-7188-8-31 (PMC3892132; doi:10.1186/1748-7188-8-31)
Supplement: Additional file 7 — Table S2. Classification performance measures in the 4CV, using (a) combined and (b) best single grammars, after vertical averaging at selected FP rate thresholds. [file 1748-7188-8-31-S7.pdf]

4CV measures

Supplementary Table 2a: Classification performance measures in the 4CV, using combined grammars, after vertical averaging, at selected FP rate thresholds

	FP rate	*Precison*	*Recall*	*F1*	*Accuracy*
c1acc	0.3	0.48	0.3	0.37	0.51
	0.5	0.52	0.58	0.55	0.54
	0.7	0.53	0.85	0.65	0.56
c2vol	0.3	0.40	0.60	0.48	0.68
	0.5	0.33	0.75	0.46	0.56
	0.7	0.30	0.92	0.46	0.45
c3vol	0.3	0.22	0.44	0.30	0.66
	0.5	0.18	0.58	0.28	0.51
	0.7	0.18	0.78	0.29	0.38
c4acc	0.3	0.12	0.33	0.17	0.66
	0.5	0.10	0.46	0.16	0.50
	0.7	0.13	0.88	0.23	0.36

Supplementary Table 2b: Classification performance measures in the 4CV, using best grammars, after vertical averaging, at selected FP rate thresholds

	FP rate	*Precison*	*Recall*	*F1*	*Accuracy*
c1acc	0.3	0.50	0.33	0.40	0.52
	0.5	0.54	0.63	0.58	0.56
	0.7	0.50	0.78	0.61	0.53
c2vol	0.3	0.39	0.58	0.47	0.67
	0.5	0.34	0.77	0.47	0.57
	0.7	0.30	0.92	0.46	0.45
c3vol	0.3	0.27	0.58	0.37	0.68
	0.5	0.24	0.83	0.38	0.55
	0.7	0.19	0.83	0.31	0.39
c4acc	0.3	0.17	0.52	0.26	0.68
	0.5	0.16	0.79	0.27	0.53
	0.7	0.15	1.00	0.26	0.38
